# Active Thermal Metasurfaces Enable Superscattering of Thermal Signatures Across Arbitrary Shapes and Thermal Conductivities

**DOI:** 10.1002/advs.202519386

**Published:** 2025-12-05

**Authors:** Yichao Liu, Yawen Qi, Fei Sun, Jinyuan Shan, Hanchuan Chen, Yuying Hao, Hongming Fei, Binzhao Cao, Xin Liu, Zhuanzhuan Huo

**Affiliations:** ^1^ Key Lab of Advanced Transducers and Intelligent Control System Ministry of Education and Shanxi Province College of Physics and Optoelectronic Engineering Taiyuan University of Technology Taiyuan 030024 China

**Keywords:** heat manipulation, thermal metasurfaces, thermal superscattering

## Abstract

The concept of superscattering is extended to the thermal field through the design of a thermal superscatterer based on transformation thermotics. A small thermal scatterer of arbitrary shape and conductivity is encapsulated within an engineered negative‐conductivity shell, forming a composite that mimics the scattering signature of a significantly larger scatterer. Crucially, the enlarged thermal scattering signature substantially exceeds that of the original small scatterer and engineered shell combined, demonstrating more than mere cross‐section amplification—an effect referred to as thermal superscattering. The amplified signature can match either a conformal larger scatterer (preserving conductivity) or a geometry‐transformed one (modified conductivity). The implementation employs a positive‐conductivity shell integrated with active thermal metasurfaces, demonstrated through three representative examples: super‐insulating thermal scattering, super‐conducting thermal scattering, and equivalent thermally transparent effects. Experimental validation shows the fabricated superscatterer amplifies the thermal scattering signature of a small insulated circular region by nine times, effectively mimicking the scattering signature of a circular region with ninefold radius. This approach enables thermal signature manipulation beyond physical size constraints, with potential applications in thermal superabsorbers/supersources, thermal camouflage, and energy management.

## Graphical abstract

This study proposes a thermal superscatterer capable of manipulating thermal scattering signatures far exceeding the actual scale of objects. Utilizing transformation thermotics and active thermal metasurfaces, the device reproduces the thermal scattering signature of the enlarged thermal scatterer. This approach provides a versatile platform for customized thermal fields and supports the application of heat management and thermal camouflage.

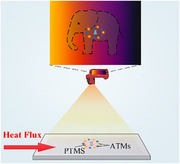

## 1 Introduction

Superscattering originates from a special optical illusion designed using transformation optics.^[^ ^1^, ^2^ ^]^ Unlike conventional scattering, superscattering refers to a phenomenon where the electromagnetic (EM) scattering cross‐section of a structure, which consists of an original EM scatterer encapsulated by a metamaterial or metasurface shell, far exceeds the geometrical cross‐section of the entire structure.^[^ ^3^, ^4^ ^]^ Moreover, this enhanced scattering cross‐section is entirely equivalent to the scattering cross‐section of a transformed object, which is obtained by applying a spatial folding transformation or other composite coordinate transformations to the original EM scatterer.^[^ ^5^ ^]^ This effect is typically achieved by encapsulating the original EM scatterer with a complementary medium pair consisting of a negative refractive index material and air, known as a superscatterer.^[^ ^1^, ^2^, ^3^, ^4^, ^5^ ^]^ It should be noted that superscattering can also refer to cases where the scattering cross‐section of a scatterer is significantly enhanced by surrounding it with plasmonic/dielectric shells^[^ ^6^ ^]^ or gain metasurfaces,^[^ ^7^ ^]^ resulting in an overall scattering cross‐section that surpasses the single‐channel limit of subwavelength structures.^[^ ^8^ ^]^ In contrast, superscatterers designed using transformation optics can produce an enhanced scattering cross‐section that is identical to that of an expanded transformed object. Consequently, superscatterers designed using transformation optics can be extended to other optical illusions, such as superabsorbers^[^ ^9^ ^]^ and invisible gateways.^[^ ^4^, ^10^ ^]^

**Figure 1:**
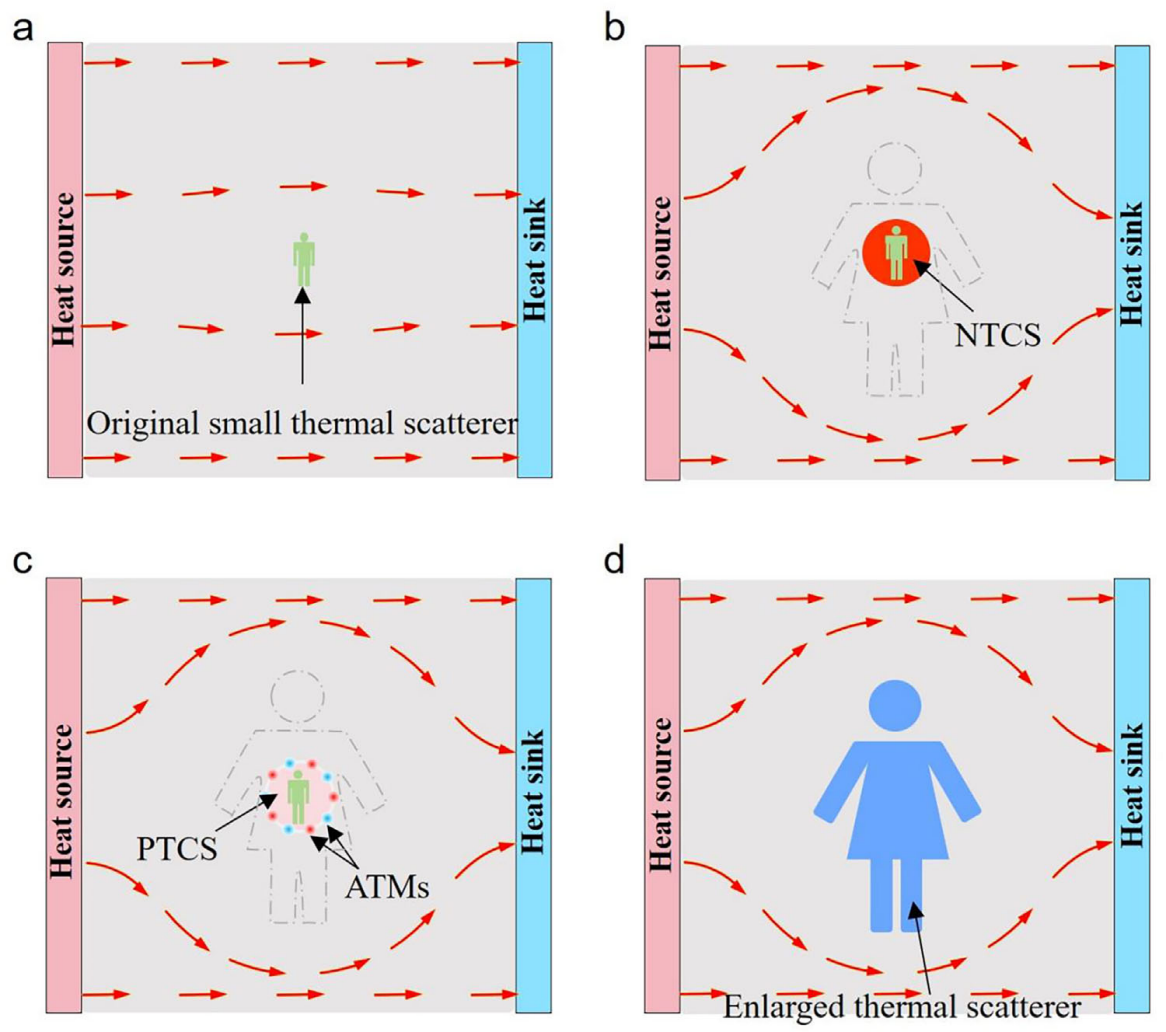
Schematic diagram of the thermal superscatterer. a) A small thermal object (indicated by a small green man) with thermal conductivity *κ*_1_ in a uniform background thermally conducting medium with thermal conductivity *κ_b_* functions as the original thermal scatterer. b) The original thermal scatterer is covered by NTCS (the red shell) in the same background thermally conducting medium, resulting in thermal superscattering. c) The original thermal scatterer is surrounded by ATMs in the same background thermally conducting medium, also resulting in thermal superscattering. d) An enlarged thermal scatterer (indicated by a large blue woman) with thermal conductivity *κ_a_* in the same background thermally conducting medium can produce the same thermal scattering signature (i.e., the same temperature distribution and heat flux distribution) as the region outside the dashed lines in (b, c). The red arrows indicate the direction of the heat flux, and the gray dashed lines in (b, c) serve as an equivalent thermal scattering cross‐section for comparison. A corresponding explanation of how the enlarged thermal scatterer in Figure 1d is generated by adding the NTCS from Figure 1b and the ATMS from Figure 1c to the small thermal scatterer in Figure 1a is provided in Movie S1 (Supporting Information).

In recent years, a similar concept has been extended to the thermal field as the thermal superscatterer, also known as the thermal magnifier^[^ ^11^ ^]^ or amplifier.^[^ ^12^ ^]^ Generally, thermal superscattering can be categorized as a specific type of thermal illusion.^[^ ^13^ ^]^ This phenomenon is primarily achieved by encapsulating an original thermal scatterer of small size and thermal conductivity *κ*_a_ (as shown in **Figure** **1**a) with an engineered shell. The resulting composite structure, formed through the integration of the original scatterer with an encapsulating engineered shell, is referred to as a thermal superscatterer. The encapsulating engineered shell with specially designed materials can be designed as a negative thermal conductivity shell (NTCS) in Figure 1b or a positive thermal conductivity shell (PTCS) together with active thermal metasurfaces (ATMs) in Figure 1c. As a result, the size of the region where the external heat flux is perturbed becomes significantly larger than that of the composite structure of the original small thermal scatterer and the engineered shell. This implies that the thermal scattering signature of the original small thermal scatterer, together with the encapsulating engineered shell, is greatly amplified. Moreover, the thermal scattering signature of the small thermal scatterer together with the engineered shell is entirely equivalent to that of an enlarged thermal scatterer with a scaled‐up size. The enlarged thermal scatterer can either preserve the thermal conductivity while maintaining conformal geometry with the original small thermal scatterer, or undergo intentional modifications to both its shape and thermal conductivity (as shown in Figure 1d). From the perspective of heat flux regulation and temperature field distribution, the temperature field outside the dashed lines in Figure 1b,c is identical to that outside the enlarged thermal scatterer in Figure 1d (also see Movie S1, Supporting Information for the explanation of how thermal super‐scattering effects are generated). In the thermal phenomena described in Figure 1a–d, the regions outside the original thermal scatterer, the enlarged thermal scatterer, and the thermal superscatterer are a uniform heat‐conducting background medium with a thermal conductivity of *κ_b_*. Furthermore, it is crucial to emphasize that the size of the enlarged thermal scattering in Figure 1d is significantly larger than the original small thermal scattering combined with the NTCS in Figure 1b and with the ATMs in Figure 1c. This is precisely why it is termed the “thermal superscattering effect,” not merely an amplification of the thermal scattering cross‐section.

With the advancement of transformation thermotics^[^ ^14^, ^15^ ^]^ and thermal metamaterials,^[^ ^16^, ^17^, ^18^, ^19^, ^20^ ^]^ many novel thermal illusion devices have been proposed, whose primary functions include reshaping the size and spatial distribution of thermal scatterers (thermal reshaper),^[^ ^12^, ^21^ ^]^ completely eliminating the thermal scattering signature of the scatterer (thermal cloaking),^[^ ^22^, ^23^, ^24^, ^25^, ^26^ ^]^ rotating or concentrating heat flux (rotator and concentrator),^[^ ^27^, ^28^, ^29^, ^30^ ^]^ removing the original thermal signature of a scatterer while generating a new one,^[^ ^13^, ^31^ ^]^ and achieving a Janus thermal function that depends on the direction of incident heat flux.^[^ ^32^, ^33^, ^34^ ^]^ While pioneering theoretical investigations have laid important groundwork for understanding thermal superscattering through analytical solutions of Laplace's equation in cylindrical coordinates,^[^ ^11^, ^12^ ^]^ several critical challenges remain to be addressed. First, existing analyses have been limited to canonical cylindrical/spherical geometries, leaving the more complex scattering behaviors of arbitrarily shaped structures unexplored. More significantly, the experimental realization of thermal superscatterers has proven challenging due to fundamental difficulties in realizing negative thermal conductivity, resulting in no experimental demonstrations reported. To further expand the range of achievable thermal conductivity, our recent research demonstrates that negative thermal conductivity can be equivalently realized by ATMs,^[^ ^35^ ^]^ which has already been successfully used to realize a long‐focus thermal lens.^[^ ^36^ ^]^ In this study, we design ATMs with precisely tailored spatial distributions and thermal output powers (as illustrated in Figure 1c) to effectively replicate the functionality of negative thermal conductivity (as depicted in Figure 1b) and subsequently demonstrate a thermal superscatterer experimentally.

## 2 Results

### 2.1 General Design Method

**Figure 2:**
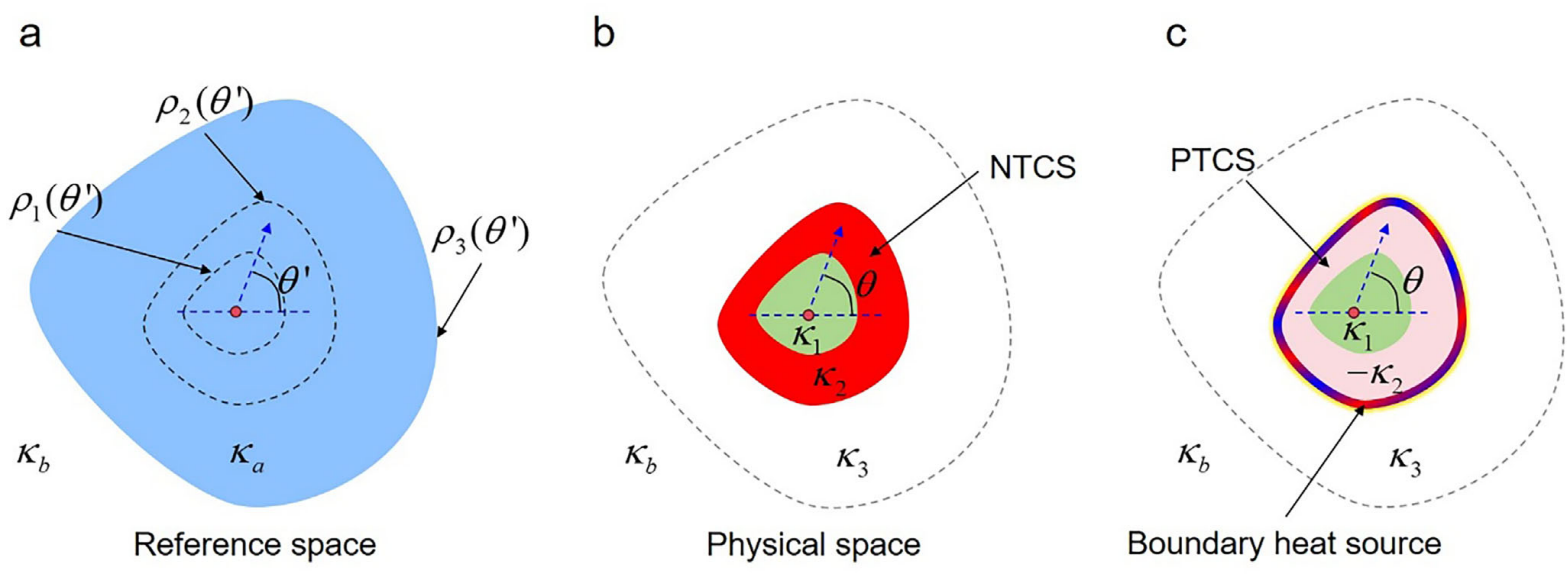
Design of the NTCS and the PTCS with a boundary heat source. a) A large thermal scatterer (colored blue) with thermal conductivity *κ_a_* in the reference space, which corresponds to the enlarged thermal scatterer in Figure 1d. b) A small thermal scatterer (colored green) with thermal conductivity *κ*_1_ covered by an NTCS (colored red) in the physical space, which is transformed from the large thermal scatterer by the coordinate transformation in Equation (1). c) The NTCS is replaced by a PTCS (colored pink) with a boundary heat source. All three cases have the same thermal scattering signature outside the boundary *ρ*_3_(*θ*). The white regions are the same background thermally conducting medium with thermal conductivity *κ_b_*.

First, we design an NTCS capable of achieving thermal superscattering in Figure 1b by transformation thermotics. The design is based on a steady‐state system described in cylindrical coordinates (*ρ*, *θ*, *z*) under a 2D configuration. Notably, the implementation follows an inverse approach, which reverses the design sequence from panels (a–d) in Figure 1. Specifically, the enlarged thermal scatterer in Figure 1d is modeled as a thermal object characterized by a thermal conductivity of *κ_a_* and a boundary defined by *ρ*_3_(*θ*). It is centrally embedded within the background material, which has a thermal conductivity of *κ_b_*, in the reference space, as illustrated in **Figure** **2**a. The original small thermal scatterer and the NTCS in Figure 1b is modeled as the thermal objects in the physical space illustrated in Figure 2b. The thermal conductivity of the original small thermal scatterer and the NTCS are *κ*_1_ and *κ*_2_, respectively, while their outer boundaries are represented by *ρ*_1_(*θ*) and *ρ*_2_(*θ*), respectively. Now, we will demonstrate how to establish the correspondence between the reference space in Figure 2a and the physical space in Figure 2b through a continuous composite coordinate transformation.

To ensure that the original small thermal scatterer encapsulated with the NTCS in the physical space (Figure 2b) can produce an identical thermal scattering signature to the enlarged thermal scatterer in the reference space (Figure 2a), a composite coordinate transformation can be employed. This transformation involves folding the boundary *ρ*_3_(*θ*) inward to *ρ*_1_(*θ*) with *ρ*_2_(*θ*) as the fixed boundary, while simultaneously compressing the region enclosed by *ρ*_3_(*θ*) into the interior of *ρ*_1_(*θ*). Note the symbols with and without primes denote the coordinates in the reference space and physical space, respectively, and the angular coordinates remain the same during the transformation, i.e., *θ* = *θ*. The detailed coordinate transformation formula can be written as:

(1) $$\left\{\begin{array}{c} \rho =\frac{\rho_{1}\left(\theta^{\prime }\right)}{\rho_{3}\left(\theta^{\prime }\right)}\rho^{\prime }\mathrm{for}\rho \leq \rho_{1}\left(\theta \right)\\ \rho =\frac{\rho_{2}{\left(\theta^{\prime }\right)}^{2}}{\rho^{\prime }}\mathrm{for}\rho_{1}\left(\theta \right)<\rho \leq \rho_{2}\left(\theta \right),\;\;\theta =\theta \prime ,\mathrm{and}\;z=z\prime \\ \rho =\rho^{\prime }\mathrm{for}\rho >\rho_{2}\left(\theta \right) \end{array}\right.$$

The transformation continuity condition requires the boundary parameters to satisfy the geometric relation: *ρ*_1_ *ρ*_3_ = *ρ*_2_2. The thermal conductivity for the small thermal scatterer *κ*_1_ within region *ρ*_<_ *ρ*_1_(*θ*), the NTCS *κ*_2_ within region *ρ*_1_(*θ*) _<_ *ρ*_<_ *ρ*_2_(*θ*), and the background *κ*_3_ within region *ρ*_>_ *ρ*_2_(*θ*), can be calculated using transformation thermotics^[^ ^37^ ^]^ (details can be found in the Experimental section):

(2) $$\begin{array}{c} \kappa_{1}=\left(\begin{array}{cc} 1+{\left[\frac{\partial \left(\rho_{1}/\rho_{3}\right)}{\partial \theta }\right]}^{2}{\left(\frac{\rho_{3}}{\rho_{1}}\right)}^{2} & \frac{\partial \left(\rho_{1}/\rho_{3}\right)}{\partial \theta }\frac{\rho_{3}}{\rho_{1}}\\ \frac{\partial \left(\rho_{1}/\rho_{3}\right)}{\partial \theta }\frac{\rho_{3}}{\rho_{1}} & 1 \end{array}\right)\kappa_{a},\;\mathrm{for}\;\rho_{<}\;\rho_{1}\left(\theta \right) \end{array}$$

(3)

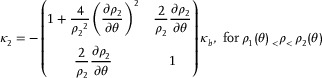

(4) $$\kappa_{3}=\kappa_{b},\;\mathrm{for}\;\rho_{>}\rho_{2}\left(\theta \right)$$

The thermal conductivity of both the NTCS described in Equation (2) and the small scatterer described in Equation (3) is typically an angular‐dependent tensor. The background thermally conducting medium outside the equivalent thermal scattering boundary (outside the dashed boxes in Figure 2b) after encapsulating the NTCS is identical to the background thermally conducting medium outside the region of the large‐sized scatterer with an outer boundary at *ρ*_3_. This is a result of the identity transformation applied beyond *ρ*_3_. Equation (4) further demonstrates that the thermally conducting material outside the boundary *ρ*_2_ in Figure 2b is identical to that outside the boundary *ρ*_3_ in Figure 2a, which means the same thermal scattering signature can be obtained with a decreased overall geometrical size.

Second, as illustrated in Figure 1c, the NTCS is equivalently replaced by a PTCS combined with a boundary heat source *q_s_* (i.e., boundary heat flux intensity). To maintain both the original temperature distribution and energy conservation, the thermal conductivity of the PTCS must satisfy *κ*_PTCS_ = −*κ*_2_, while the boundary heat source is determined by *q_s_* = −2*q_n_*.^[^ ^35^] Here, *q_n_* represents the normal component of heat flux at the NTCS boundary, adhering to the sign convention where inward‐directed fluxes toward the NTCS are defined as positive.

### 2.2 Two Special Cases with Simplified Material Parameters

Considering the complexity of the material parameters of the small scatterer and the engineered shell that display inhomogeneity and anisotropy in the absence of specific selection of the three boundaries, we focus on examining two special cases to achieve further simplification of these material parameters in Equations (2) and (3). The first case involves the conformal enlargement of the geometric region of the original small thermal scatterer with arbitrary thermal conductivity while keeping the thermal conductivity unchanged. In this case, the original small thermal scatterer and the enlarged thermal scatterer are homothetic. Specifically, it includes three examples: super‐insulating thermal scattering, super‐conducting thermal scattering, and equivalent thermally transparent effect. The second case involves simultaneously enlarging and reshaping the thermal scattering signature of the original small thermal scatterer with intentionally modified thermal conductivity. Specifically, if the original small thermal scatterer is made of adiabatic material, the enlarged thermal scatterer can also maintain adiabatic properties under coordinate transformation.

**Figure 3:**
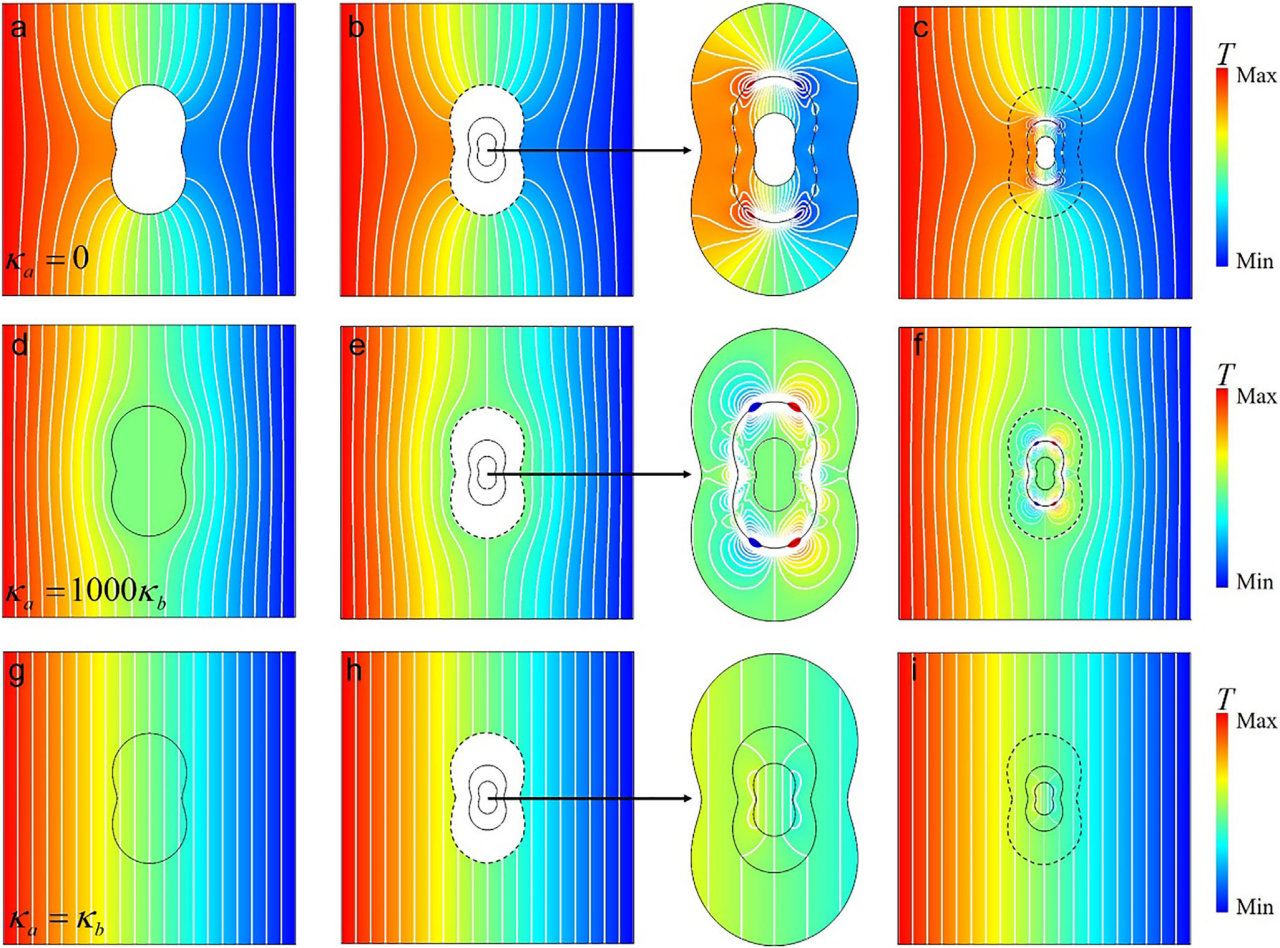
Simulation results for conformal shapes. Simulated temperature distributions for peanut‐shaped large scatterers with different thermal conductivity: a) *κ_a_*  = 0, d)*κ_a_*  = 1000*κ_b_*, and g)*κ_a_*  = *κ_b_*. In panels b, e, h), the small scatterers exhibit the same thermal conductivity as the large thermal scatterers and are covered by the same NTCS. For enhanced clarity, the regions within the boundary *ρ*_3_(*θ*) are enlarged and presented as insets. In c, f, i), PTCS and boundary heat sources are used to achieve the corresponding thermal superscattering effect, which is the same as that in (b, e, h).

In the first case, the shapes of the original thermal scatterer (indicated by the boundary *ρ*_1_(*θ*)), the engineered shell (indicated by the boundary *ρ*_2_(*θ*)), and the enlarged thermal scatterer (indicated by the boundary *ρ*_3_(*θ*)) are conformal. Their conformal nature implies that the ratio *ρ*_1_(*θ*)/*ρ*_3_(*θ*) is independent of the angular coordinate *θ*. Consequently, the partial derivative ∂(*ρ*_1_(*θ*)/*ρ*_3_(*θ*))/∂*θ* = 0, which leads to *κ*_1_ = *κ_a_* through Equation 2. This implies that if the shapes of the small thermal scatterer and the enlarged thermal scatterer are conformal, the thermal conductivity *κ_a_* of the enlarged thermal scatterer in the reference space of Figure 2a is identical to the thermal conductivity *κ*_1_ of the small thermal scatterer in the physical space of Figure 2b,c. In other words, the thermal superscatterer only magnifies the geometrical size of the thermal scattering signature without altering the material or shape of the scatterer. Under this condition, we simulate three cases where the small thermal scatterer is filled with an adiabatic material (*κ_a_* = 0), a highly conductive material (*κ_a_* = 1000*κ_b_*), and a background thermally conductive material (*κ_a_* =*κ_b_*), respectively (see **Figure** **3**).

Figure 3a shows the simulated temperature distribution when the large thermal scatterer with *κ_a_* = 0 is exposed to a uniform thermal flux. Figure 3b illustrates the temperature distribution when replacing the large thermal scatterer with a smaller thermal scatterer of the same thermal conductivity, and an NTCS whose thermal conductivity follows Equation (3). The resulting temperature distribution closely resembles that of the large thermal scatterer. In Figure 3c, the NTCS is replaced by a corresponding PTCS, as well as a boundary heat source. Note only the boundary heat source for boundary *ρ*_2_(*θ*) needs to be considered when *κ_a_* = 0, as the normal boundary heat flux along boundary *ρ*_1_(*θ*) is zero. In contrast, when *κ_a_*  ≠ 0, both boundaries must be taken into account. In this example, with the aid of thermal super‐scatterers shown in Figure 3b,c, adiabatic materials occupying a smaller area encapsulated with the engineered shells can achieve the same thermal shielding effect as those covering a larger area for the external background region, thereby realizing the super‐insulating thermal scattering effect (or super‐thermal shielding effect). In this case, the isothermal line distribution in the external region *ρ>ρ*_3_ shown in Figure 3b,c is identical to that in Figure 3a, where heat flux cannot penetrate the same area. This creates an illusion that external heat flow cannot enter the region *ρ<ρ*_3_, while in reality the structure remains capable of sensing external heat flow‐achieving the “thermally inaccessible yet thermally perceptive” phenomenon. Therefore, this configuration can be used to create virtual thermal insulation boundaries. Moreover, in this example, the geometric dimensions of the engineered shell *ρ*_2_ are smaller than those of the virtual adiabatic boundary *ρ*_3_. This creates a transitional zone between the engineered shell and the virtual insulation boundary that maintains the same thermal conductivity as the background material. This region appears thermally inaccessible from the external perspective while actually permitting heat flux penetration ‐precisely embodying the essential meaning of “super” in the term super‐scattering phenomenon.

In Figure 3d, the thermal conductivity of the enlarged thermal scatterer is chosen as a high value, i.e., *κ_a_*  = 1000*κ_b_*. Two cases demonstrate that a small thermal scatterer, enveloped by an NTCS in Figure 3e and a PTCS together with a suitably designed boundary heat source in Figure 3f, respectively, can both achieve a temperature distribution similar to that of the large thermal scatterer with the same high thermal conductivity in Figure 3d. In this example, with the aid of thermal super‐scatterers shown in Figure 3e,f, materials with high thermal conductivity occupying a smaller area encapsulated with the engineered shells can achieve the same good heat conduction effect as those covering a larger area for the external background region, thereby realizing the super‐conducting thermal scattering effect. This means that high thermal conductivity materials occupying a smaller area (*ρ<ρ*_1_), when encapsulated with the engineered shells (*ρ*_1_ *<ρ<ρ*_2_), can be used to achieve the same heat flow attraction and convergence effects as those of materials with the same thermal conductivity occupying a larger area (*ρ<ρ*_3_). Notably, the geometric dimension of the engineered shell *ρ*_2_ is intentionally designed with a geometric dimension smaller than that of the enlarged high‐conductivity thermal scatterer *ρ*_3_. The intervening space between them (*ρ*_2_ *<ρ<ρ*_3_) is occupied by a low thermal conductivity background material *κ_b_*, which is far smaller than the thermal conductivity of the enlarged thermal scatterer *κ_a_*  = 1000*κ_b_*. Therefore, this configuration effectively mimics a virtual high‐conductivity boundary, enabling applications such as efficient heat energy collectors (effectively increasing the heat concentration cross‐sectional area of the thermally conductive material), integration of thermal sinks as super‐thermal absorbers, or embedding heat generators as super‐thermal sources.

More interestingly, if the large thermal scatterer vanishes (*κ_a_*  = *κ_b_*) in Figure 3g, we can still design an NTCS in Figure 3h and a PTCS together with a suitably designed boundary heat source in Figure 3i, respectively, to achieve an equivalent thermally transparent effect. In this case, although the original small thermal scatter, when encapsulated with the engineered shells in Figure 3h,i, does not affect the distribution of the temperature field and heat flux in the external region *ρ>ρ*_3_. Notably, in this configuration, the annular region between the engineered functional shell and the enlarged thermal scatterer, i.e., *ρ*_2_<*ρ*<*ρ*_3_, also preserves the original thermal state, maintaining both the temperature field and heat flux distribution. However, through elaborate design of the boundary heat sources along boundaries *ρ*_2_ and *ρ*_1_, we can achieve redistribution of the temperature field and heat flux inside boundary *ρ*_2_(*θ*). That is, it is thermally transparent to the external thermal background, and the gradient distribution of the internal temperature field can be adjusted, thus enabling local thermal management without external disturbance. Note that changing the thermal conductivity of the large/small scatterer will not influence the material parameter of the NTCS and PTCS, which is only determined by the thermal conductivity of the background and the profile of the boundary *ρ*_2_(*θ*).

Although the first three case studies adopt a specific geometric configuration, the proposed method demonstrates universal applicability to thermal superscatterers of arbitrary shapes. As detailed in Note S1 (Supporting Information), we have successfully designed superscatterers with alternative geometries‐including triangular and rectangular configurations‐through systematic numerical simulations and theoretical analyses.

**Figure 4:**
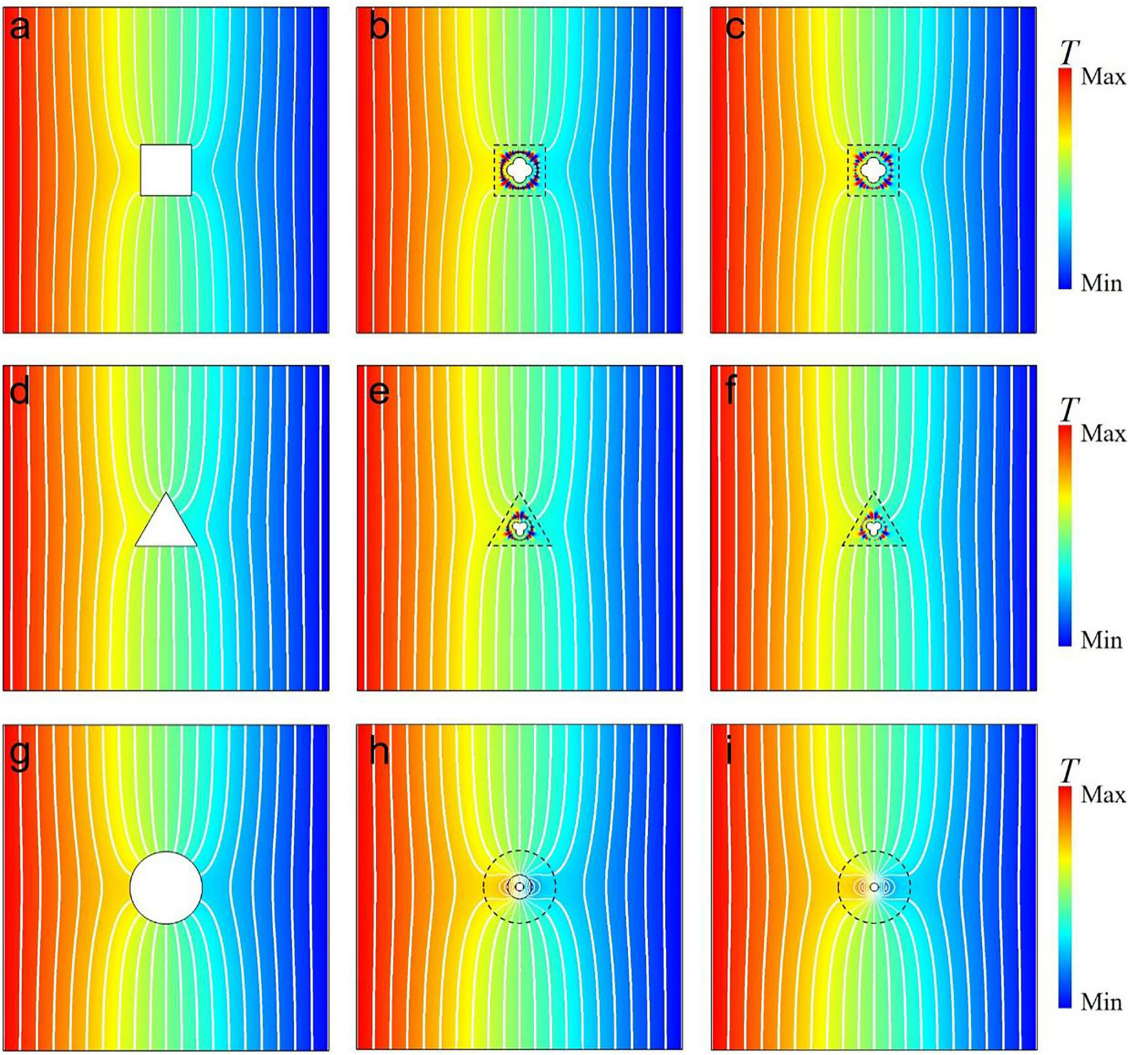
Simulation results for boundary *ρ*_2_(*θ*) = const. a, d, g) are the simulated temperature distributions for large scatterers with different shapes. b, e, h) are the corresponding superscatterers that consist of small scatterers and NTCSs. c, f, i) are the corresponding superscatterers that consist of small scatterers and circular boundary heat sources. For enhanced clarity of the temperature distribution within the superscatterers, the isotherms inside the boundary *ρ*_3_(*θ*) are removed in (b, c, e, f). Note that the small thermal scatterers are designed as adiabatic materials, which leads to the corresponding enlarged thermal scatterers exhibiting adiabatic characteristics according to Equation (2).

The second case corresponds to *ρ*_2_(*θ*) = const, indicating that the engineered shell now possesses a circular outer boundary with a radius of *R*_2_. In this case, the thermal conductivity of the NTCS region can be simplified to be the negative of the background material, i.e., *κ*_2_ = ‐*κ_b_*, which can be derived from Equation (3) by setting the partial derivative ∂*ρ*_2_(*θ*)/∂*θ* = 0. Then, the modified thermal conductivity of enlarged thermal scatterers *κ*_a_ can be designed through the transformation in Equation (2). Such modification can be intentionally achieved by designing the ratio *ρ*_1_(*θ*)/*ρ*_3_(*θ*) and the thermal conductivity of smaller thermal scatterers *κ*_1_. As a special case, when the small thermal scatterers are adiabatic materials (i.e., *κ*_1_ = 0), Equation (2) reveals that the corresponding enlarged thermal scatterers also exhibit adiabatic characteristics (with unchanged material thermal conductivity *κ*_a_ = 0). Meanwhile, the small thermal scatterers often exhibit geometrical shapes that differ from those of the enlarged thermal scatterers, except for a circular shape. For example, the small four‐petal‐shaped scatterer in **Figure** **4**b,c can generate identical thermal scattering signatures to the enlarged square in Figure 4a, while the small three‐petal‐shaped scatterer in Figure 4e,f produces identical thermal scattering features to the enlarged triangle in Figure 4d.

Figure 4a,d,g presents the simulation results for three large thermal scatterers with different shapes, each having a thermal conductivity of *κ_a_* = 0. When these large thermal scatterers in Figure 4a,d,g are replaced by the small thermal scatterers combined with engineered shells, the corresponding temperature distributions associated with the NTCS are illustrated in Figure 4b,e,h, whereas the corresponding temperature distributions with boundary heat sources are shown in Figure 4c,f,i. The close resemblance to the large scatterers depicted in Figure 4a,d,g demonstrates that the thermal superscattering effect can be achieved using only a boundary heat source at *ρ*_2_(*θ*) = const, where the PTCS is replaced by the background materials (i.e., *κ*_PTCS_ = *κ_b_*). For the cases of superconducting thermal scattering and equivalent thermal transparency, in addition to modifying the thermal conductivity of the small scatterer to align with that of the enlarged scatterer, it is also necessary to introduce a boundary heat source at *ρ*_1_. This means that subsequent ATM design should be implemented on both the *ρ*_1_ and *ρ*_2_ boundaries.

These two specific cases demonstrate that the material parameters of both the small thermal scatterer and the PTCS can be greatly simplified when the enlarged thermal scatterer is thermally insulated, shares a conformal profile with the small thermal scatterer, or the *ρ*_2_(*θ*) possesses a circular profile. Even in the absence of these conditions, the anisotropic material parameters and the boundary heat source distribution can still be determined using our general methodology, as detailed in Note S1.3 (Supporting Information). In addition to comparing the temperature field distributions and isotherms to analyze the consistency between the superscatterer and the enlarged thermal scatterer, we further analyze both a generalized thermal superscatterer and the circular thermal superscatterer based on the average temperature discrepancy. The detailed discussion is provided in Note S1.4 (Supporting Information).

### 2.3 Design of the ATMs Array

**Figure 5:**
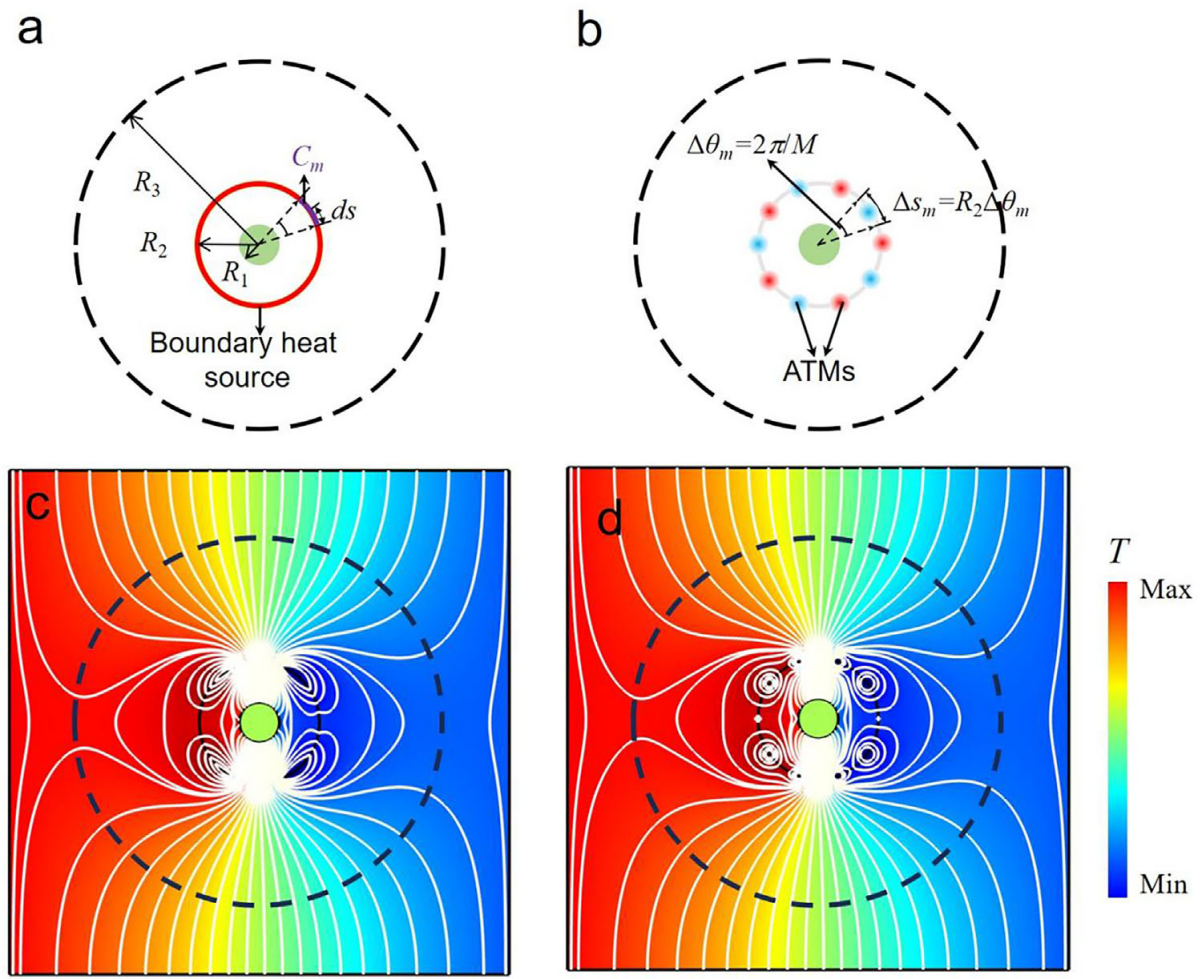
Design diagram of the ATMs array. a) Structural diagram of the thermal superscatterer with boundary heat source. The red line represents the boundary heat source. b) Structural diagram of the thermal superscatterer with ATMs. Red/blue dots represent ATMs. In (a, b), all materials except for the central adiabatic region have the same thermal conductivity as the background material, which is represented by the white areas. The spacing angle between adjacent ATMs is Δ*θ* = 36°. And Δ*s* represent the corresponding arc length for each ATMs. Since the small thermal scatterer, engineered shell, and enlarged thermal scatterer all exhibit circular geometries in this case, their boundary profiles reduce to constant radial functions independent of *θ*: *ρ*_1_(*θ*) = *R*_1,_ *ρ*_2_(*θ*) = *R*_2_ *ρ*_3_(*θ*) = *R*_3_. c) Simulated result of a thermal superscatterer with a continuous boundary heat source. d) Simulated temperature distribution and isotherms for a thermal superscatterer with 10 ATMs. In (c, d), the white lines represent the isotherms. The black dashed small and big circles represent the boundary where heat source or ATMs are located *ρ*_2_(*θ*) = *R*_2_ and the virtual enlarged boundary *ρ*_3_(*θ*) = *R*_3_, respectively.

To experimentally realize the superscattering effect, an array of ATMs should be designed to equivalently achieve the boundary heat source. For ease of fabrication and processing, this study uses the small circular thermal scatterer filled with adiabatic materials in Figure 4i as an example to demonstrate the design methodology for replacing boundary heat sources with ATMs and to validate the approach through subsequent experimental verification. Here, the boundary heat source at the boundary *ρ*_2_(*θ*) = *R*_2_ in **Figure** **5**a is discretized into *M* curve segments (with equal angular spacing Δ*θ* = 2*π*/*M* and equal arc length Δ*s* = Δ*θR*_2_), where an ATM is placed at the midpoint of each segment, as shown in Figure 5b. The heat power of each ATM is obtained by integrating the boundary heat flux density *q_s_* over the corresponding curved segment:^[^ ^35^ ^]^

(5) $$Q_{m}=\int_{C_{m}}q_{s}ds$$

where *C_m_* represents the *m*‐th curved segment on the circular arc of radius *R*_2_, and d*s* is the arc length differential element on *C_m_*. Since the simulated boundary heat source exhibits oscillatory behavior along *ρ*_2_(*θ*), the sampling theorem requires at least two discretization points per oscillation period. Our approach discretizes each periodic region of the heat source distribution into positive (heat‐releasing) and negative (heat‐absorbing) segments, with integration performed separately within each segment to obtain the discretized heat source distribution. Increasing the number of sampling points, i.e., increasing the discretization number *M*, would bring the external temperature field of the discretized thermal superscatterer closer to that of the continuous boundary heat source case. However, considering the practical constraints of experimental fabrication, *M* should not be chosen excessively large. Moreover, when *M* = 10, the uniformly distributed ATMs with a 36° angular interval are located almost identically to those obtained from strictly discretizing the positive and negative segments of the boundary heat source. They already provide sufficiently good performance. Considering these factors, the configuration with *M* = 10 and ATMs uniformly distributed starting from 0° is selected for subsequent numerical and experimental studies. Further details regarding the influence of the discretization number *M* on superscatterer performance are provided in Note S2 (Supporting Information).

Before conducting experiments, the effectiveness of the method by replacing the boundary heat sources in Figure 5a with ATMs in Figure 5b is validated through numerical simulations. For the configurations in Figure 5a,b under identical thermal flux incidence from left to right (with boundary conditions specified in the numerical settings), the simulated temperature fields and isotherms are shown in Figure 5c,d, respectively. The simulated temperature distribution and isotherms outside the boundary *R*_2_ for the case using discrete ATMs in Figure 5d are fully consistent with those for the case using continuous boundary heat sources in Figure 5c. The simulated results verify that replacing the boundary heat sources in Figure 5a by discretizing the boundary into *M* = 10 equal segments and allocating the total heat flux of each segment to an ATM positioned at its midpoint in Figure 5b can effectively replicate the effects of continuous boundary heat sources, thereby enabling the super‐insulating thermal scattering effect in Figure 4i. Therefore, the ATM configuration shown in Figure 5b will be adopted for subsequent experimental validation on the super‐insulating thermal scattering effect.

## 3 Experimental Verification

**Figure 6:**
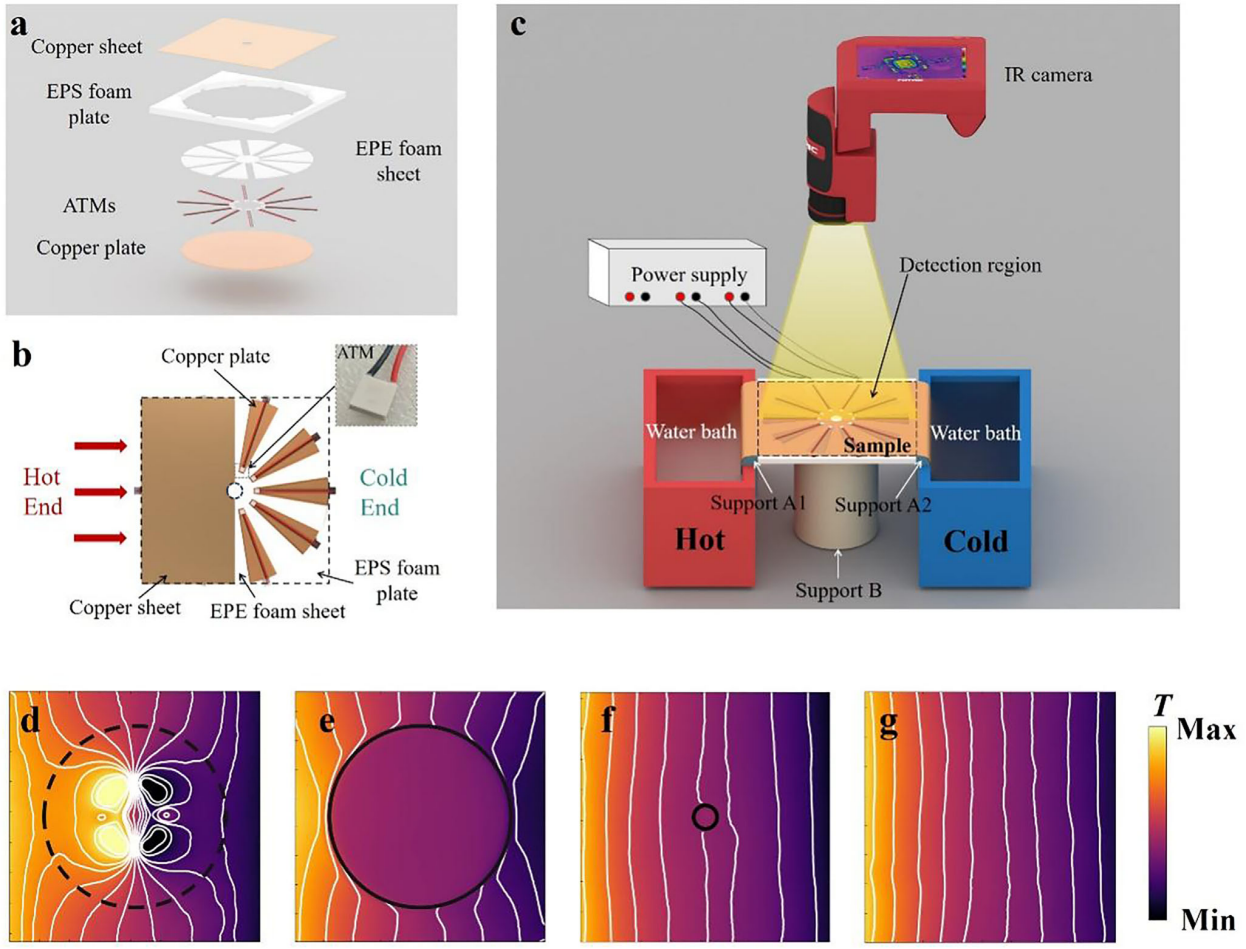
Experimental setup and results. a) Assembly details of the sample. b) Schematic diagram of the designed thermal superscatterer with 10 ATMs (top view). The dashed line represents the complete shape of the copper sheet in the detection region. 10 ATMs are evenly distributed along a circle with radius of *R*_2_ = 30 mm. c) Schematic diagram of the experimental setup. The detection region is indicated by the dashed lines. The water baths on both sides provide constant temperature heat sources for the experiment. d–g) Each depicts the temperature distribution and isotherms as measured in the detection region. (d) The temperature distribution and isotherms of the thermal superscatterer. A dashed circle is plotted for comparison. (e) There is a 90 mm‐radius circular hole at the center of the copper sheet. (f) There is a 10 mm‐radius circular hole at the center of the copper sheet. (g) The detection region is entirely made up of a copper sheet. Temperature distributions and isotherms depicted in (e–g) all serve as references for the effect of thermal superscatterer.

By utilizing the design shown in Figure 5b, the sample is fabricated in **Figure** **6**a,b, and the experimental measurement system is established in Figure 6c. The sample preparation and assembly process are given in the subhead Sample Preparation of Experimental Section and Note S3 (Supporting Information), shown in Figure 6a. Here, the upper copper sheet serves as the background thermal conduction medium, while the bottom copper plate acts as a heat dissipation sink. Expanded Polyethylene (EPE) foam sheet and expanded polystyrene (EPS) foam are used for supporting and simulating thermal insulation, respectively. 10 semiconductor cooling plates are utilized as the ATMs, which can generate thermal output power as designed by Equation (5) using tunable loading current^[^ ^38^ ^]^ and are evenly fixed on the circle *R*_2_ = 30 mm. The assembled sample is shown in Figure 6b, positioned within the detection region of the experimental measurement system illustrated in Figure 6c. A mechanically rigid material (polylactic acid fiber with a thermal conductivity of 0.13 W·m^−1^·K^−1^ and a height of 20 cm) serves as Support B to ensure alignment between the upper surface of the ATMs and the upper surfaces of supports A1 and A2 (polylactic acid fiber with a thermal conductivity of 0.13 W·m^−1^·K^−1^). The function of Supports A1 and A2 on the sidewalls of the two water baths is to allow the two ends of the copper sheet to be smoothly immersed in the water along the rounded edges of the supports. This configuration also provides sufficient space beneath the copper plate for heat convection, enabling the copper plate's temperature to stabilize near ambient temperature. The experiment is conducted in an enclosed room with a constant temperature *T*_room_ = 300.65 K.

An electric constant‐temperature water bath (labeled as “hot”, colored red) serves as the heat source, while a large insulated water bath (labeled as “cold”, colored blue) acts as the heat sink. Throughout the experiment, the temperatures of the heat source and heat sink remain nearly constant at 320 and 287 K, respectively. This ensures that the left and right boundaries of the detection region maintain stable, constant temperatures of 307 and 297 K. The temperature difference between the heat source and heat sink induces a heat flux in the detection region, which aligns with the simulation results presented in Figures 3, 4, 5. To minimize the influence of natural convection, the experimental detection region is enclosed with EPS foam boards. Subsequently, experimental measurements are conducted on the assembled sample, which is positioned within the detection region and observed by the infrared camera (FOTRIC 288). Based on the required heat power *Q*_m_ calculated from Equation (5) for each ATM, the corresponding current for each port of the DC power supply is set according to the calculated value.^[^ ^35^, ^38^ ^]^ After energizing the ATMs, a 30‐s waiting period is required until the temperature field distribution observed by the infrared camera stabilizes, i.e., the system reaches a steady state. Then, the temperature distribution of the detection region is recorded by the infrared camera. Due to the neglect of factors in the initial current calculations, such as environmental convection disturbances and sample preparation errors, appropriate adjustments should be made to the applied current of the DC power supply. To address this discrepancy, the proposed methodology involves comparing the temperature distribution obtained from the initial experimental measurements with the simulation results in Figure 5d. Through systematic analysis of isotherm distribution disparities, the applied currents for relevant ATMs are fine‐tuned, ensuring close alignment between the experimental and simulated isotherm distributions. For more details regarding experimental measurement, please refer to Note S4 and Movie S2 (Supporting Information).

The measured temperature distribution of the original small thermally insulated circular region with radius *R*_1_ = 10 mm, when encapsulated with precisely designed 10 ATMs (functioning as the engineered shell) in Figure 6d, shows consistency with the corresponding simulation result in Figure 5d. For comparison, reference measurements are also conducted for three additional cases: a copper sheet with a large circular thermally insulated region (*R*_3_ = 90 mm) in Figure 6e, a copper sheet with a small circular thermally insulated region (*R*_1_ = 10 mm) in Figure 6f, and an intact copper sheet without any thermally insulated region in Figure 6g.

Compared with the case without any thermal scatter in Figure 6g, the measured result in Figure 6f demonstrates that the small thermal scatterer exhibits a negligible impact on both the temperature distribution and the isotherms. However, when the small circular thermally insulated region with radius *R*_1_ = 10 mm is scaled up by a factor of nine, the enlarged thermal scatterer with radius *R*_3_ = 90 mm significantly alters both the temperature distribution and the isotherms in Figure 6e. Notably, the temperature and isotherm distributions in Figure 6d are nearly consistent with those in Figure 6e. This indicates that the thermal scattering signature of the larger thermally insulated circular region in Figure 6e can be effectively replicated by the original small thermally insulated circular region encapsulated with precisely engineered ATMs in Figure 6d. Therefore, the experimental results conclusively demonstrate that a small thermal insulated scatterer enclosed by precisely engineered ATMs can successfully emulate a larger thermal insulated scatterer, thereby experimentally validating the super‐insulating thermal scattering effect. Notably, the super‐insulating thermal scattering effect achieved here enables the creation of “virtual thermal insulation boundaries”, where external heat flux perceives an enlarged insulated region, while the actual structure retains compact dimensions. Such capability opens avenues for applications in thermal camouflage, where localized heat management can be achieved without altering macroscopic material layouts.

## 4 Discussion

In summary, the concept of superscattering has successfully been extended to the thermal field by designing and experimentally validating a thermal superscatterer based on transformation thermodynamics and ATMs. The method for the realization of thermal superscattering effect involves no negative thermal conductivity materials, thus making its realization feasible. The design framework supports three distinct thermal illusions: super‐insulating scattering (mimicking enlarged adiabatic regions and achieving the “thermally inaccessible yet thermally perceptive” by virtual thermal insulation boundaries), super‐conducting scattering (emulating large high‐conductivity domains by virtual high‐conductivity boundaries), and equivalent thermal transparency (masking internal thermal perturbations). The experimental results validate that a small thermally insulated circular region encapsulated with an array of 10 precisely engineered ATMs can effectively magnify the thermal scattering cross‐section of an insulated circular region by a factor of 9. The thermal scattering cross‐section can be increased without limit in theory, but in practice, the amplification factor is limited by the excessive power consumption of the ATMs and the difficulties of heat dissipation. The method in this study can be extended to other applications, such as super thermal insulation, thermal invisibility gateways, thermal superabsorbers, and thermal supersources. Moreover, the method may be further improved by combining it with other promising techniques. On the one hand, the heat diffusion invariant methods^[^ ^39^ ^]^ provide a direct way to calculate the required heat capacity from a predetermined thermal conductivity, thereby extending device functionality to transient states. On the other hand, deep learning^[^ ^40^, ^41^, ^42^, ^43^ ^]^ and conformality‐assisted tracing^[^ ^44^ ^]^ can facilitate self‐adaptive control, free‐form shape design, and extension to multiphysics field regulation.

## 5 Experimental Section

### Derivation of Thermal Conductivity

The derivation is performed in the cylindrical coordinate system, where the metric tensor in the reference space and physical space can be expressed as *g*′ = *diag*{1, ρ′^2^,1}, γ = *diag*{1, ρ^2^,1}, respectively. The thermal conductivity in the cylindrical coordinate system can be expressed as:

(6) $$\kappa_{\mathrm{cyl}.}=\frac{\sqrt{detg^{\prime }}}{\sqrt{det\gamma }}\frac{Jg^{-1}J^{T}}{detJ}\kappa_{\mathrm{ref}.}$$

where *J* is the Jacobian matrix of the transformation and *κ*_ref_ is the thermal conductivity in the reference space. When using the normalized coordinate basis, the thermal conductivity can be written as:

(7) $$\kappa =\Lambda \kappa_{cyl.}\Lambda^{T}$$

where Λ = *diag*{1, ρ, 1}. Equation (7) can be used to derive thermal conductivity for different regions with different transformations in Equation (1).

### Derivation of Thermal Conductivity

The numerical simulations were all conducted by commercial software COMSOL Multiphysics 5.6 with the license number 9406999 (https://www.comsol.com), which is based on the finite element method. The 2D solid heat transfer module with a steady‐state solver was selected to simulate the temperature field distributions in all the simulations. The free tetrahedral meshing with automatically generated mesh grid is used.

For all the simulations in this study, the left boundary was set to a constant high temperature of 307.65 K, the right boundary was set to a constant low temperature of 297.65 K, and the background thermal conductivity was set to 400 W·m^−1^·K^−1^. In Figure 3, *ρ*_3_(*θ*)/*ρ*_2_(*θ*) = *ρ*_2_(*θ*)/*ρ*_1_(*θ*) = 2. The side length of the square *l*_1_ in Figure 4a–c, the side length of the triangle *l*_2_ in Figure 4d–f, and the radius of the outer circle *R*_3_ in Figure 4g–i, has the following relationship with the circle *ρ*_2_(*θ*) = *R*_2_, i.e., *l*_1_ = $2\sqrt{2}$ *R*_2_, *l*_2_ = $3\sqrt{3}$ *R*_2_, *R*_3_ = 3*R*_2_.

### Sample Preparations

First, a round copper plate with a radius of 125 mm and a thickness of 3 mm was prepared for the heat dissipation of ATMs. Then 10 points were marked on the copper plate to indicate the positions of the ATMs. These 10 points were arranged in a circle *ρ*_2_(*θ*) = *R*_2_ with the equal spacing angle Δ*θ* = 36° as shown in Figure 5b. The semiconductor cooling plate (TECooler Technology, model HT009022, size 9.8 mm × 9.8 mm × 2.59 mm) was selected to be utilized as ATMs. The 10 ATMs were positioned at the designated locations on the copper plate. Then, the remaining space between the ATMs was filled with Expanded Polyethylene (EPE) foam sheet that has a thickness of 2 mm and a thermal conductivity of 0.05 W·m^−1^·K^−1^. An expanded polystyrene (EPS) foam board with a thickness of 10 mm and a thermal conductivity of 0.035 W·m^−1^·K^−1^ was used to encase the lateral air region surrounding the copper plate in the detection region. Both EPE foam sheet and EPS foam board serve to reduce heat exchange and provide support for the copper sheet, with their upper surfaces aligned with the upper surface of the ATMs. A copper sheet with a size of 550 mm × 250 mm × 0.1 mm and featuring a circular hole with a radius of *R*_1_ = 10 mm was positioned above the ATMs. To reduce thermal resistance, thermal conductive silicone grease was used to fill the air gaps between the ATMs and both the copper plate beneath and the copper sheet above it. To ensure a uniform high surface emissivity, blackbody paint was applied to the upper surface of the copper sheet. For additional details regarding sample fabrication and experimental setup, refer to Note S3 (Supporting Information).

## Conflict of Interest

The authors declare no conflict of interest.

## Supporting information



Supporting Information

Supplemental Movie 1

Supplemental Movie 2

## Data Availability

The data supporting the findings of this study are available from the corresponding authors upon reasonable request.

## References

[1] T. Yang , H. Y. Chen , X. D. Luo , H. Ma , Optics Exp. 2008, 16, 18545. 10.1364/oe.16.018545 18958133

[2] W. H. Wee , J. B. Pendry , New J. Phys. 2009, 11, 073033.

[3] H. X. Xu , G. Wang , K. Ma , T. J. Cui , Adv. Opt. Mater. 2014, 2, 572.

[4] C. Li , X. Meng , X. Liu , F. Li , G. Fang , H. Chen , C. T. Chan , Phys. Rev. Lett. 2010, 105, 233906. 21231465 10.1103/PhysRevLett.105.233906

[5] C. F. Yang , J. J. Yang , M. Huang , J. H. Peng , G. H. Cai , Comput. Mater. Sci. 2010, 49, 820.

[6] C. Qian , X. Lin , Y.i Yang , X. Xiong , H. Wang , E. Li , I. Kaminer , B. Zhang , H. Chen , Phys. Rev. Lett. 2019, 122, 063901. 30822094 10.1103/PhysRevLett.122.063901

[7] C. Qian , Y.i Yang , Y. Hua , C. Wang , X. Lin , T. Cai , D. Ye , E. Li , I. Kaminer , H. Chen , Nat. Commun. 2022, 13, 4383. 35902584 10.1038/s41467-022-32067-9 PMC9334305

[8] Z. C. Ruan , S. H. Fan , Phys. Rev. Lett. 2010, 105, 013901. 20867445 10.1103/PhysRevLett.105.013901

[9] J. Ng , H. Y. Chen , C. T. Chan , Opt. Lett. 2009, 34, 644. 19252579 10.1364/ol.34.000644

[10] G. D. Bai , F. Yang , W. X. Jiang , Z. L. Mei , T. J. Cui , Appl. Phys. Lett. 2015, 107, 153503.

[11] S. Yang , L. J. Xu , J. P. Huang , J. Appl. Phys. 2019, 125, 055103.

[12] X. He , L. Z. Wu , Appl. Phys. Lett. 2014, 105, 221904.

[13] R. Hu , S. L. Zhou , Y. Li , D. Y. Lei , X. Luo , C. W. Qiu , Adv. Mater. 2018, 30, 1707237. 10.1002/adma.201707237 29665110

[14] L. J. Xu , J. P. Huang , in Transformation Thermotics and Extended Theories, Inside and Outside Metamaterials, Springer Nature, Singapore, 2023.

[15] S. Guenneau , C. Amra , D. Veynante , Opt. Express 2012, 20, 8207. 22453491 10.1364/OE.20.008207

[16] S. Yang , J. Wang , G. L. Dai , F. B. Yang , J. P. Huang , Phys. Rep. 2021, 908, 1.

[17] R. Hu , W. Xi , Y. Liu , K. Tang , J. Song , X. Luo , J. Wu , C.‐W. Qiu , Mater. Today 2021, 45, 120.

[18] Z. Zhang , L. Xu , T. Qu , M. Lei , Z.‐K. Lin , X. Ouyang , J.‐H. Jiang , J. Huang , Nat. Rev. Phys. 2023, 5, 218.

[19] F. Yang , Z. Zhang , L. Xu , Z. Liu , P. Jin , P. Zhuang , M. Lei , J. Liu , J.‐H. Jiang , X. Ouyang , F. Marchesoni , J. Huang , Rev. Mod. Phys. 2024, 96, 015002.

[20] Y. Li , W. Li , T. Han , X.u Zheng , J. Li , B. Li , S. Fan , C.‐W. Qiu , Nat. Rev. Mater. 2021, 6, 488.

[21] H. Chen , F. Sun , B.o Wang , Y. Liu , Z. Chen , Y. Yang , Int. J. Therm. Sci. 2022, 176, 107506.

[22] R. Schittny , M. Kadic , S. Guenneau , M. Wegener , Phys. Rev. Lett. 2013, 110, 195901. 23705719 10.1103/PhysRevLett.110.195901

[23] H. Y. Xu , X. H. Shi , F. Gao , H. D. Sun , B. L. Zhang , Phys. Rev. Lett. 2014, 112, 054301. 24580599 10.1103/PhysRevLett.112.054301

[24] Y. G. Ma , Y. C. Liu , M. Raza , Y. D. Wang , S. L. He , Phys. Rev. Lett. 2014, 113, 205501. 25432046 10.1103/PhysRevLett.113.205501

[25] Y. Liu , X. Ma , K. Chao , F. Sun , Z. Chen , J. Shan , H. Chen , G. Zhao , S. Chen , Opto‐Electron. Sci. 2024, 3, 230027.

[26] S. He , R. Zhang , J. Liang , Opto‐Electron. Adv. 2024, 7, 240211.

[27] S. Guenneau , C. Amra , Opt. Express 2013, 21, 6578. 23482229 10.1364/OE.21.006578

[28] T. Han , J. Zhao , T. Yuan , D. Y. Lei , B. Li , C.‐W. Qiu , Energy Environ. Sci. 2013, 6, 3537.

[29] S. Narayana , Y. Sato , Phys. Rev. Lett. 2012, 108, 214303. 23003263 10.1103/PhysRevLett.108.214303

[30] H. Chen , Y. Liu , F. Sun , Q. Sun , X. Wu , R. Sun , Laser Photonics Rev. 2024, 18, 2400488.

[31] T. C. Han , X. Bai , J. T. L. Thong , B. W. Li , C. W. Qiu , Adv. Mater. 2014, 26, 1731. 24497430 10.1002/adma.201304448

[32] Y. S. Su , X. W. Zhang , Y. G. Sun , J. Xiong , Appl. Phys. Lett. 2022, 120, 141901.

[33] Y. Su , Y. Li , T. Yang , T. Han , Y. Sun , J. Xiong , L. Wu , C.‐W. Qiu , Adv. Mater. 2021, 33, 2003084. 10.1002/adma.202003084 33306245

[34] K. Chao , F. Sun , H. Chen , Y. Liu , Z. Chen , X. Ma , Z. Chen , J. Wang , AIP Adv. 2023, 13, 105304.

[35] Y. Liu , K. Chao , F. Sun , S. Chen , H. Dai , H. Chen , Adv. Mater. 2023, 35, 2210981. 10.1002/adma.202210981 37060549

[36] Y. Liu , F. Sun , S. He , Opt. Expr. 2016, 24, 5683. 10.1364/OE.24.005683 27136765

[37] J. Huang , in Theoretical Thermotics, Transformation Thermotics and Extended Theories for Thermal Metamaterials, Springer, Singapore, 2020.

[38] D. M. Nguyen , H. Xu , Y. Zhang , B. Zhang , Appl. Phys. Lett. 2015, 107, 121901.

[39] L. Xu , P. Zhuang , F. Yang , S. Yang , C. Wang , G. Dai , J. Huang , C.‐W. Qiu , Phys. Rev. Lett. 2025, 135, 067103. 40864935 10.1103/cny8-szn7

[40] Y. Wang , W. Sha , M. Xiao , C. Qiu , L. Gao , Adv. Mater. 2023, 35, 2302387. 10.1002/adma.202302387 37394737

[41] P. Jin , L. Xu , G. Xu , J. Li , C.‐W. Qiu , J. Huang , Adv. Mater. 2024, 36, 2305791. 10.1002/adma.202305791 37869962

[42] Z. Li , J. Sun , Y. Fan , Y. Jin , Q. Shen , M. Trusiak , M. Cywinska , P. Gao , Q. Chen , C. Zuo , Opto‐Electron. Sci. 2023, 2, 220023.

[43] Y. Chen , F. Zhang , Z. Dang , X. He , C. Luo , Z. Liu , P. Peng , Y. Dai , Y. Huang , Y. Li , Z. Fang , Opto‐Electronic Science 2023, 2, 220019.

[44] L. Xu , G. Dai , F. Yang , J. Liu , Y. Zhou , J. Wang , G. Xu , J. Huang , C.‐W. Qiu , Nat. Comput. Sci. 2024, 4, 532. 38982225 10.1038/s43588-024-00660-1

