# Supplementary material for: Active Thermal Metasurfaces Enable Superscattering of Thermal Signatures Across Arbitrary Shapes and Thermal Conductivities
Source: Adv Sci (Weinh). 2025 Dec 5;13(10):e19386. doi: 10.1002/advs.202519386 (PMC12915085; doi:10.1002/advs.202519386)
Supplement: Supplementary file 1 — Supporting Information [file ADVS-13-e19386-s001.docx]

Supplementary Information

**Active Thermal Metasurfaces Enable Superscattering of Thermal Signatures Across Arbitrary Shapes and Thermal Conductivities**

Yichao Liu, Yawen Qi, Fei Sun^*^, Jinyuan Shan, Hanchuan Chen, Yuying Hao_,_ Hongming Fei, Binzhao Cao, Xin Liu, Zhuanzhuan Huo

**1 Thermal Superscatterers of Different Geometries**

**1.1 Design of Triangular Superscatterer**

According to the design method described in the main text, we calculate the thermal conductivity in each region based on the NTCS region division shown in Figure S1(a). The outer boundary of the NTCS region, denoted as *ρ*_2_(*θ*), can be expressed as follows:

$\left\{\begin{matrix} \rho_{2}^{(1)}(\theta)=l_{2}\frac{\sqrt{1+tan^{2}\theta}}{\sqrt{3}+\tan\theta} \\ \rho_{2}^{(2)}(\theta)=l_{2}\frac{\sqrt{1+tan^{2}\theta}}{\sqrt{3}-\tan\theta} \\ \rho_{2}^{(3)}(\theta)=l_{2}\frac{\sqrt{1+\frac{1}{tan^{2}\theta}}}{2} \end{matrix}\right.$. (S1)

*l*_2_ is the distance from the center to the vertex of the NTCS triangular outer boundary which described by *ρ*_2_(*θ*). The numerical simulation of the triangular superscatterer in a two-dimension is conducted. We set the thermal conductivity of each NTCS region to the calculated values and choose air as the material for both the original small and enlarged thermal scatterers. The boundary conditions of *ρ*_1_(*θ*) are set to thermal insulation. The power of the introduced boundary heat source is obtained from the numerical simulation result of triangular thermal superscatter with the NTCS and used for further numerical simulation. Figures S1(b)–S1(e) show the numerical simulation results for the original small triangular thermal scatterer, the enlarged triangular thermal scatterer, the triangular thermal superscatterer with the NTCS, and the triangular thermal superscatterer realized by the boundary heat source, respectively. By comparing the temperature distributions and isotherms among these cases, the functionality of the triangular thermal superscatterer is verified.

**1.2 Design of Square Superscatterer**

In the design of the square superscatterer, we also performed a regional division, dividing the NTCS region into four regions and calculating the thermal conductivity for each region. The specific division of regions is shown in Figure S2(a). The outer boundary of the NTCS, described by *ρ*_2_(*θ*), can be expressed as:

$\left\{\begin{matrix} \rho_{2}^{(1)}(\theta) =\frac{l_{2}}{2cos\theta} \\ \rho_{2}^{(2)}(\theta)=\frac{l_{2}}{2sin\theta} \\ \rho_{2}^{(3)}(\theta)=-\frac{l_{2}}{2cos\theta} \\ \rho_{2}^{(4)}(\theta)=-\frac{l_{2}}{2sin\theta} \end{matrix}\right.$. (S2)

*l*_2_ denotes the side length of the square boundary corresponding to the outer boundary of the NTCS region. The original small square thermal scatterer has a side length of *l*_1_ = 0.02 m. The side length of the small square thermal scatterer is *l*_2_ = 0.04 m. The enlarged square thermal scatterer has a side length of *l*_3_ = 0.08 m. The thermal conductivity of the background material is *κ_b_*. The NTCS region is divided into four sub - regions, with thermal conductivities of *κ*_2_^(1)^, *κ*_2_^(2)^, *κ*_2_^(3)^ and *κ*_2_^(4)^, respectively. Two - dimensional numerical simulations are conducted for the following four cases: original small square thermal scatterer, enlarged square thermal scatterer, square thermal superscatterer with NTCS, and square thermal superscatterer achieved by boundary heat source. The simulation results demonstrate that the proposed design approach can also yield effective square thermal superscatterer.

**1.3 Design of a generalized superscatterer without simplified parameters**

In this case, we choose a circle with *R*_1_ = 0.01 m as the boundary profile of the small thermal scatterer, i.e., *ρ*_1_(*θ*) = 0.01. The boundary of the enlarged thermal scatterer described by *ρ*_3_(*θ*), can be expressed as:

$\rho_{3}(\theta) =\frac{l_{3}}{\text{2max(}\left|cos \theta\right|, \left|sin \theta\right|)}$

*l*_3_ = 0.06 m denotes the side length of the square boundary of the enlarged thermal scatterer. Due to the transformation continuity condition of *ρ*_1_*ρ*_3_ = *ρ*_2_^2^, *ρ*_2_(*θ*) can be calculated, and its profile is shown in Figure S3a. The thermal conductivity of the background material is *κ_b_*. The thermal conductivities of the small thermal scatterer (*κ*_1_) and the PTCS (-*κ*_2_) can be obtained using Equation 2 and Equation 3 in the main text. The corresponding boundary heat sources along *ρ*_1_ and *ρ*_2_ can be determined. Two-dimensional numerical simulations are conducted for the following four cases: original small thermal scatterer, enlarged thermal scatterer, the thermal superscatterer with NTCS, and the thermal superscatterer achieved by boundary heat source. The simulation results verify that the proposed design method without any parameter simplification can still effectively achieve the desired function. As shown in Figure S3(b), when no simplification is applied, the thermal conductivity distributions of both the NTCS region and the small thermal scatterer exhibit anisotropy and inhomogeneity.

**1.4 Discussion of the Average Temperature Discrepancy for Different Thermal Superscatterer**

To further verify the consistency between the thermal scattering signature of the thermal superscatterer and that of the enlarged thermal scatterer, the average temperature discrepancy Δ*T* can be calculated in the region of *ρ* > *ρ*₃ for comparison. The Δ*T* can be expressed as:

$\Delta T=\sqrt{\frac{\text{∬}_{S} (T-T_{0})^{2}dS}{S}}$. (S4)

In the equation, *T* denotes the temperature distribution for a thermal superscatterer in the region of *ρ* > *ρ*₃, *T*_0_ represents the temperature distribution of the enlarged thermal scatterer of the corresponding region, and *S* is the area of the corresponding region. We calculated the Δ*T* for the circular and a generalized thermal superscatterer implemented with the NTCS, the boundary heat source, and ATMs, respectively. The generalized thermal scatterer features the square profile of *ρ*₃(*θ*) and the circular profile of *ρ*_1_(*θ*), as discussed in the Supplementary Note 1.3. The Δ*T* values for both the generalized and circular thermal superscatterers with boundary heat sources are presented in Table S1. These values are extremely small (<0.0013 K) when compared to the background temperature range (293.15 K to 303.15 K), confirming the high consistency between the external temperature fields of the superscatterer with boundary heat sources and the enlarged scatterer. The Δ*T* values for both the generalized and circular thermal superscatterers with ATMs are presented in Table S1. When the boundary heat source is replaced by ATMs, Δ*T* increases, indicating that the discretization introduces a deviation in the external temperature field. For the generalized thermal superscatterer, the increase of Δ*T* is more pronounced, because both the thermal conductivity distribution and boundary heat source distribution are more complex in this case. Nevertheless, relative to the overall temperature field, the Δ*T* remains sufficiently small to demonstrate that the thermal scattering signatures exhibited by the thermal superscatterers with ATMs are consistent with that of the enlarged thermal scatterer.

**2 Impact of Discretization Number *M* on Thermal Superscatterer Performance**

To realize the circular thermal superscatterer in experiments, the boundary heat source is discretized. Specifically, the boundary *C*_2_ is divided into *M* curved segments, with each segment represented by an ATM located at its center, effectively replacing the continuous boundary heat source. Since the number of ATMs influences the performance of the superscatterer, numerical simulations are conducted for *M* = 4, 6, 8, 10, 12, 14 and 16. In addition, the average temperature discrepancy Δ*T* can be calculated in the region of *ρ* > *ρ*₃ according to the Equation S4 for comparison. In this case, the Δ*T_M_* is the average temperature discrepancy Δ*T* for different discretization number *M*, and *T*_0_ represents the temperature distribution of the enlarged thermal scatterer in corresponding region. As shown in Figure S3, the Δ*T_M_* approaches zero as *M* increases. It indicates the performance of circular thermal superscatterer improves as *M* increases. When *M* = 10, the average temperature discrepancy is ∆*T*_10_ = 0.97×10^-2^ K.

**3 Sample Preparation and Pre-experiment Preparation**

**3.1 Sample Preparation**

(1) A circular copper plate with a radius of 125 mm and a thickness of 3 mm is fabricated using laser cutting method. Mark a concentric circle with a radius of 30 mm on the circular copper plate, and mark 10 points with an equal interval angle of 36° on the circle. These points correspond to the positions where the centers of the ATMs would be placed during the experiment.

(2) The 10 ATMs (TECooler Technology, model HT009022, size 9.8 mm×9.8 mm×2.59 mm) are placed at the marked points on the copper plate. A thermally conductive silicone grease with a thermal conductivity of 6 W/(m·K) is filled between the ATMs and the copper plate. This method ensures contact between the ATMs and the copper plate, effectively reducing thermal resistance. The wires of the ATMs are fixed on the surface of the copper plate with tape for ensuring them stable.

(3) Fill the copper plate surface—excluding the ATMs and their wires—with Expandable Polyethylene (EPE) foam sheet, and fasten them firmly with tape to minimize heat exchange between ATMs. The EPE foam sheet has a thickness of 2 mm and a thermal conductivity of 0.05 W/(m·K). Level the EPE foam sheet top flush with the ATMs top surfaces to support the copper sheet laid over them and ensure the sample structure stays stable.

(4) Cut the Expanded Polystyrene (EPS) foam board to 280 mm × 280 mm × 9 mm. At its center, cut a 125 mm‑radius circle, then punch ten small holes corresponding with the position of the ATMs so their wires can pass through. Fit the prepared EPS foam board around the copper plate, then organize the ATMs wires and guide them through the corresponding small holes. Align the top surface of the EPS foam broad with those of the ATMs and the EPE form sheet.

(5) Fabricate three copper sheets with size 550 mm × 250 mm × 0.1 mm. Cut a 10 mm‑radius circular air hole at the center of the first sheet and a 90 mm‑radius circular air hole at the center of the second use laser cutting method, and leave the third sheet intact. In the experiment, the detection region is the central 250 mm × 250 mm region on each sheet. Uniformly apply blackbody paint to the detection regions of the all three copper sheets to ensure their top surfaces exhibit uniform and high emissivity. Place the sheet with the 10 mm‑radius circular air hole over the ATMs, apply the thermally conductive silicone grease between the ATMs and the copper sheet to assemble the thermal superscatterer sample for experiment.

**3.2 Pre-experiment Preparation**

(1) Place the assembled sample on Support A, keeping its bottom of the copper plate 20.5 cm above the ground. This can provide sufficient space beneath the copper plate for natural air convection and effective heat dissipation. In the system, the copper plate functions as a heat sink. It absorbs or releases heat from the low surfaces of the ATMs to keep the ATMs work well.

(2) Prepare the heat sources. Place a temperature constant water bath on the left side and set its temperature to 320.65 K to serve as the high temperature heat source. On the right side, place an insulated water bath filled with water at 287.65 K as the cold source. Inside both the water bath and the insulated water bath, install Support B so that its top surfaces align with the upper surfaces of the EPS foam board, EPE foam sheets and ATMs—providing a level base for the top copper sheet. Keep the copper sheet flat, immerse its ends into the water on both sides, and secure them properly to ensure the stability of the system.

(3) Organize the ATMs wires and connect each one to the corresponding output of the DC power supply. To ensure stable and safe connections, wrap the wire joints with insulating tape.

(4) Surround the experimental measurement area with EPS foam boards to minimize the impact of air convection.

(5) During the comparative measurements of the three copper sheets, place a 280 mm × 280 mm EPS foam board between the supports B in the constant temperature water bath and the insulated water bath. And adjust the height of support A so that the top surface of the EPS foam board aligns with those of Support B, providing a stable platform for the reference copper sheet. Use tape to secure the EPS foam board. Place the three copper sheets—one intact, one with a 10 mm‑radius central circular air hole, and one with a 90 mm‑radius central circular air hole—on the EPS foam board. Immerse both ends of each sheet in the water baths and fix them in place before starting the measurement.

1. **Details of Experimental Measurements**

(1) Preliminary adjustment of the input current for each ATMs. In general, the input current is the most critical operating parameter for directly controlling the ATMs. The power applied for each ATM in the simulation corresponds to the required cooling/heating capacity of the TE Cooler. Based on the cooling/heating capacity, the theoretical input current for each ATM can be calculated using Equation S4. In the experiment, turn on the DC power supply and set each output channel to constant-current mode. Then adjust the output current of each channel to match its corresponding theoretical value. After all currents have been adjusted, turn off the power supply. The theoretical current values are calculated under a linear approximation, considering only the linear term in Equation S5, and are shown in Table S2. Due to the unconsidered nonlinear effects, environmental convection and other factors, the actual input current is significantly higher than the theoretical current value.

$\left\{\begin{matrix} Q_{L}=\alpha IT_{L}\text{-}\frac{1}{2}I^{2}R \text{- }\kappa_{T}(T_{H}\text{-}T_{L}) \\ Q_{H}=\alpha IT_{H}+\frac{1}{2}I^{2}R \text{-} \kappa_{T}(T_{H}\text{-}T_{L}) \end{matrix}\right.$ (S5)

*Q_L_* and *Q_H_* represent the cooling power and heating power of the ATM, respectively. *α* is the Seebeck coefficient of the TE Cooler. *I* and *R* denote the input current and the internal resistance of the ATM, respectively. *κ_T_* is the thermal conductivity of the TE Cooler. *T_L_* and *T_H_* correspond to the temperatures of the cold side and hot side of the ATM, respectively.

(2) Add water to the constant temperature water bath and turn on its power supply. Then, add water at 287.65 K to the insulated water bath. Once the water in the constant temperature water bath reaches the preset temperature, wait for about 20 minutes for the system to stabilize. The left boundary temperature of the detection region is at 307.65 K, and the right boundary temperature is at 297.65 K.

(3) Fix the IR camera by a tripod at an appropriate height and adjust the angle of the lens to ensure the detection region at the center of the IR camera’s display.

(4) After completing Step 2 and confirming the system reaches a steady state, turn on the IR camera and begin recording the surface temperature distribution within the detection region. Switch on the DC power supply to power each ATMs through its corresponding output. Wait about 30 seconds until the temperature field in the detection region reach stable state. Then use the IR camera to capture the temperature distribution. Turn off the power supply and allow the system to return to room temperature state.

(5) Analyze the experimental data and adjust the input current values for the ATMs. Process the experimental data obtained in Step 4 to obtain the isothermal contours of the measured temperature field. Compare this temperature distribution with the numerical simulation and the experimental result of temperature distribution of the enlarged thermal scatterer. Based on the comparison, adjust the input currents of the ATMs and repeat the experimental steps to refine the experimental result. Continue this process until the experimental results align with the expected distribution. Record the final modified current values in Table S1. In the experiment, several factors can lead to discrepancies between the theoretical and modified current values, including:

(i) Nonlinear effects. According to the performance curves of the TE Cooler, when the temperature difference between the cold and hot sides is 0 K, the cooling/heating capacity is approximately linear with respect to the input current in the low-current range. However, as the input current increases, this relationship gradually becomes nonlinear due to internal Joule heat and material. In addition, when a temperature difference exists between the cold and hot sides of the ATM, a larger temperature difference causes the nonlinear behavior to appear at a lower input current.

(ii) Unavoidable fabrication errors during sample preparation. Thermal contact resistance naturally exists at the interface between materials. The fabrication errors may significantly increase the thermal contact resistance, thereby affecting the effective heat transfer.

(iii) Difficulty of heat dissipation. Due to limited heat dissipation conditions, the performance of ATMs that do not receive timely and rapid heat dissipation will be affected.

(iv) The influence of air convection. Air convection introduces an additional heat exchange at the sample surface, which is not considered in the simulation.

Therefore, the influence of these factors on the temperature field necessitates appropriate adjustments to the input current in order to achieve the expected performance of the thermal superscatterer. During this adjusting process, the operating mode of each ATM—whether cooling or heating—remains unchanged; however, the input current typically needs to be increased to compensate for the aforementioned disturbances. According to Equation S4, for the same required thermal capacity, the input current needed for cooling is higher than that for heating. Moreover, the ATMs operating in the cooling mode generally require larger adjustments to their input currents. Due to the theoretical input current calculations without consideration of nonlinear effects, there is a noticeable discrepancy between the theoretical and modified current values. These factors collectively contribute to the discrepancy between the theoretical current values and the modified current values.

(6) To clearly verify the function of the thermal superscatterer, perform comparative experimental measurements on three copper sheets: an intact one, one with a 10 mm-radius central circular air hole, and one with a 90 mm-radius central circular air hole. After completing step 2, the system reaches the steady state, then use the IR camera to record the temperature distribution within the detection region of each copper sheet.


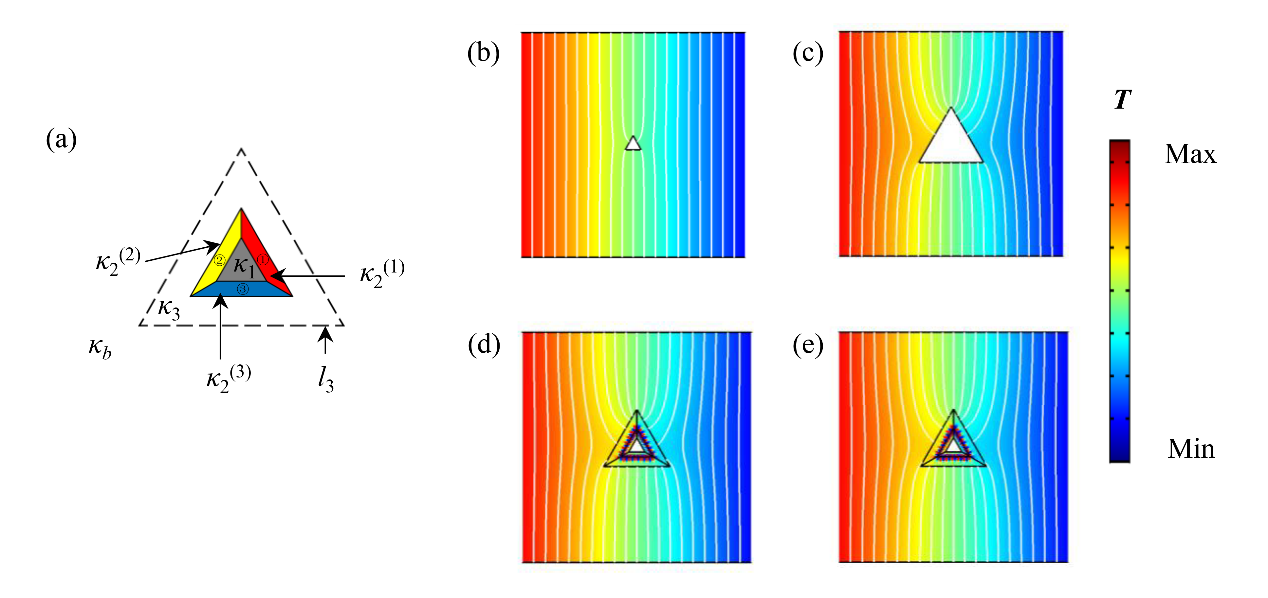


Supplementary Figure S1 **|** Schematic and simulation results of triangular thermal superscatterer. (b)-(e) Simulated temperature distributions and isotherms. Black lines in (d) and (e) mark the enlarged thermal scatterer's boundary and divide the NTCS region into three regions. The boundaries of the original small thermal scatterer, the outer of the NTCS, and the enlarged thermal scatterer, described by *ρ*_1_(*θ*), *ρ*_2_(*θ*), and *ρ*_3_(*θ*) respectively, are all equilateral triangles, with *l*_1_ = 0.01 m, *l*_2_ = 0.02 m, and *l*_3_ = 0.04 m. (a) Schematic of the triangular thermal superscatterer, with three regions designed for different thermal conductivities *κ*_2_^(1)^, *κ*_2_^(2)^ and *κ*_2_^(3)^. The area outside the large scatterer is the background material with thermal conductivity *κ_b_*; (b) Original small triangular thermal scatterer; (c) Enlarged triangular thermal scatterer; (d) Triangular thermal superscatterer with NTCS; (e) Triangular thermal superscatterer achieved by boundary heat source.


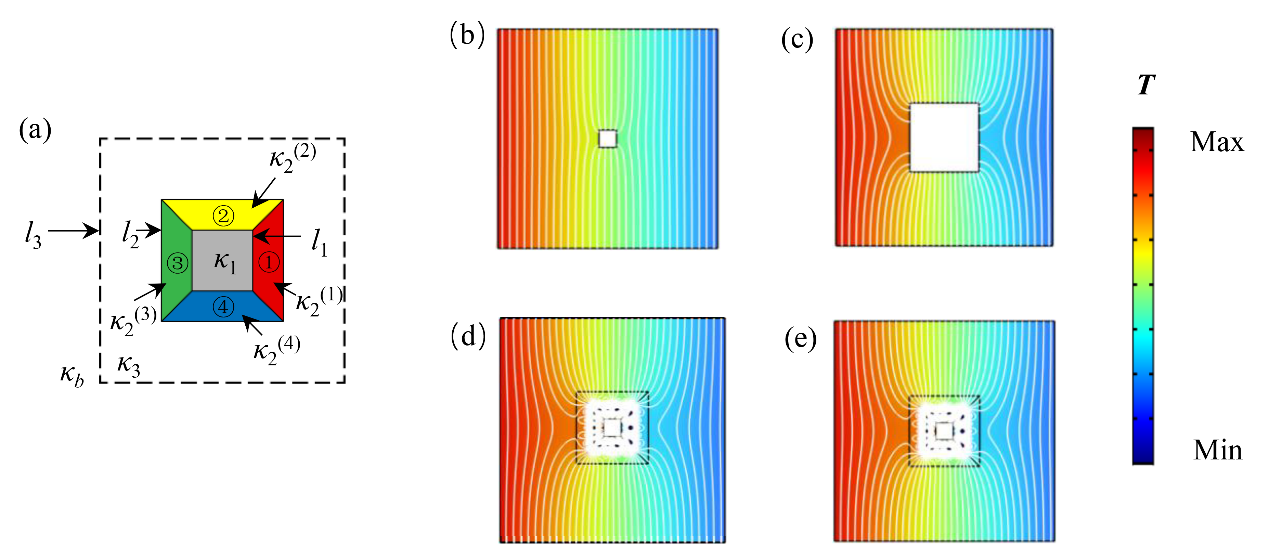


Supplementary Figure S2 **|** Structural schematic and simulation results of square thermal superscatterer. (b)-(e) show the temperature distributions and isotherms of corresponding numerical simulation. For comparison, the enlarged square thermal scatterer's boundary is marked with a black dashed line in (d) and (e). (a) Structural schematic of a square thermal superscatterer. For the square thermal superscatterer with NTCS, a original small square thermal scatterer with side length *l*_1_ = 0.02 m is enclosed by a NTCS with outer side length *l*_2_ = 0.04 m. The boundary of the enlarged thermal scatterer has a side length of *l*_3_ = 0.08 m. Different thermal conductivities *κ*_2_^(1)^, *κ*_2_^(2)^, *κ*_2_^(3)^, and *κ*_2_^(4)^ are calculated for each region. The thermal conductivity of background material is *κ*_b_; (b) A original small square thermal scatterer; (c) Enlarged square thermal scatterer; (d) Square thermal superscatterer with NTCS; (e) Square thermal superscatterer achieved by boundary heat source.


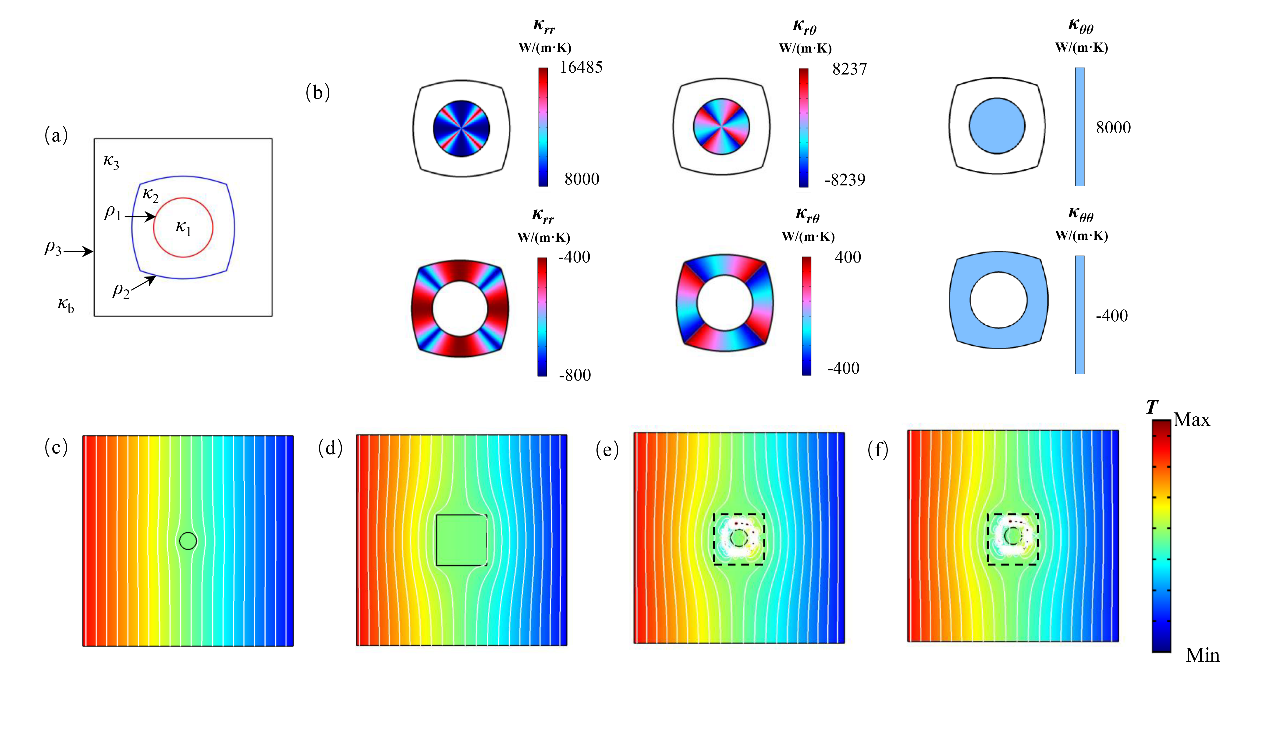


Supplementary Figure S3 **|** Structural schematic, distributions of thermal conductivity tensor components and simulation results of a generalized thermal superscatterer. (a) Structural schematic of a generalized thermal superscatterer. An original small circular thermal scatterer with radius *R*_1_ = 0.01 m and the enlarged square thermal scatterer with side length *l*_3_ = 0.06 m. The geometric relationships in the structural diagram satisfy *ρ*_1_*ρ*_3_ = *ρ*_2_^2^. The thermal conductivities of the original thermal scatterer, the NTCS region and the annular region between *ρ*_2_ and *ρ*_3_ are *κ*_1_, *κ*_2_ and *κ*_3,_ respectively. The thermal conductivity of background material is *κ*_b_. (b) show the distributions of the thermal conductivity tensor components *κ_rr_*, *κ_rθ_* and *κ_θθ_*, respectively. Since the thermal conductivity tensor in this case is symmetric, *κ_rθ_* = *κ_θr_*. These components are expressed in an orthonormal basis aligned with the directions of the cylindrical coordinates. (c)-(f) show the temperature distributions and isotherms of the corresponding numerical simulations. For comparison, the boundary of the enlarged square thermal scatterer is marked with a black dashed line in (e) and (f). (c) an original small circular thermal scatterer; (d) enlarged square thermal scatterer; (e) generalized thermal superscatterer with NTCS; (f) generalized thermal superscatterer achieved by boundary heat source.


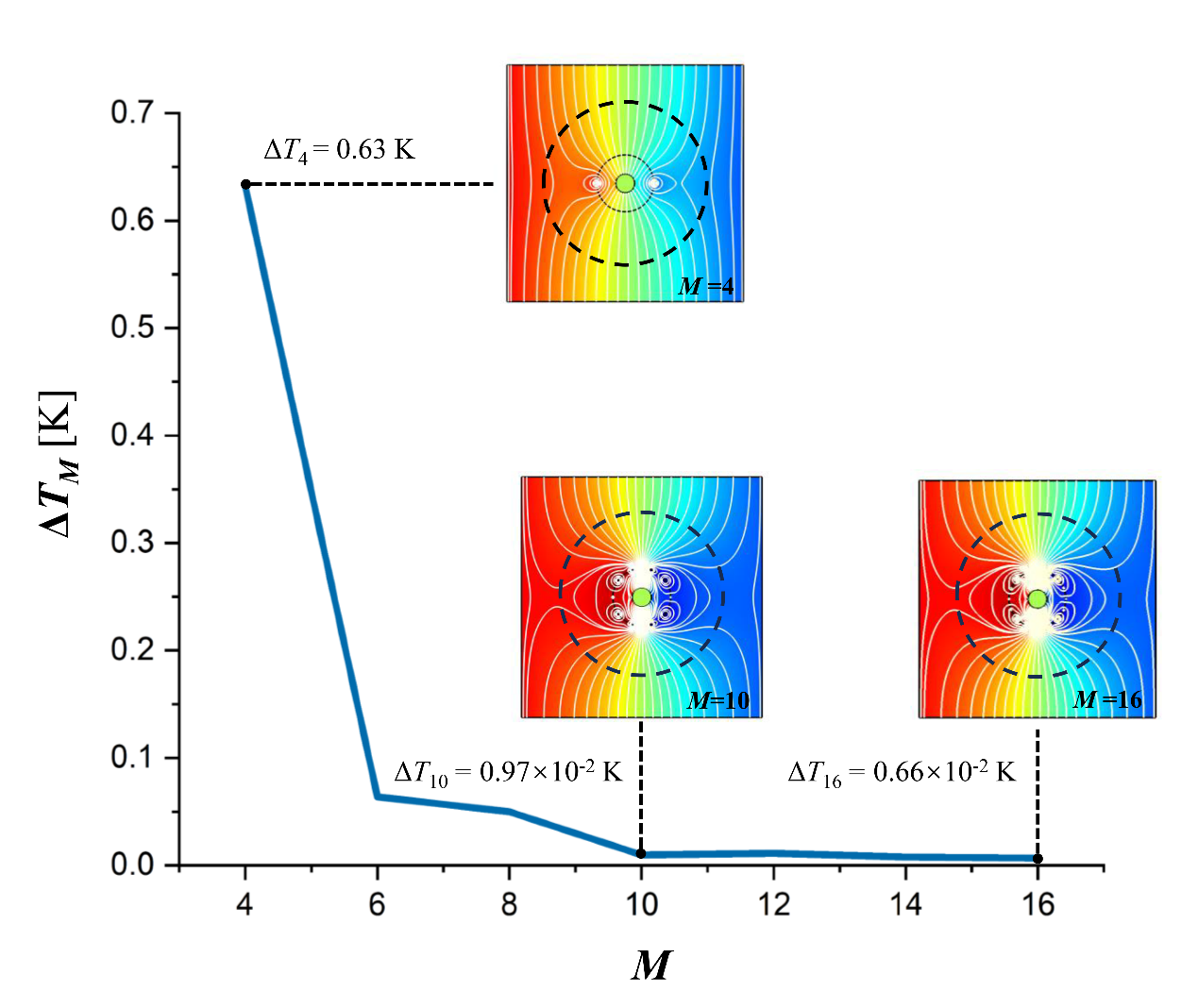


Supplementary Figure S4 **|** The average temperature discrepancy Δ*T_M_* of the thermal superscatterers with different discretization numbers *M*. The temperature distributions, isotherms and the values of Δ*T_M_* of circular thermal superscatterers with *M* = 4, 10 and 16.

Table S1 Δ*T* of the generalized thermal superscatters and circular thermal superscatterers

| Type of the thermal superscatterer | Δ*T* [K] |
| --- | --- |
| Generalized thermal superscatterer with NTCS | 9.34×10^-4^ |
| Generalized thermal superscatterer with boundary heat source | 1.26×10^-3^ |
| Generalized thermal superscatterer with ATMs | 3.27×10^-2^ |
| Circular thermal superscatterer with NTCS | 7.39×10^-4^ |
| Circular thermal superscatterer with  boundary heat source | 8.23×10^-4^ |
| Circular thermal superscatterer with ATMs | 1.07×10^-2^ |

Table S2 Current values and position angles of ATMs

| Number | Angle of ATMs [deg] | Theoretical current value[A] | | Modified current value[A] |
| --- | --- | --- | --- | --- |
| 1 | 0 | | 0.055 | 0.542 |
| 2 | 36 | | -0.316 | -1.587 |
| 3 | 72 | | -0.723 | -1.763 |
| 4 | 108 | | 0.686 | 0.984 |
| 5 | 144 | | 0.300 | 0.889 |
| 6 | 180 | | -0.058 | -0.618 |
| 7 | 216 | | 0.300 | 1.014 |
| 8 | 252 | | 0.686 | 1.088 |
| 9 | 288 | | -0.723 | -1.425 |
| 10 | 324 | | -0.316 | -1.239 |

(*The sign of the theoretical and modified current values indicates the mode of the ATM: a positive current corresponds to heating, while a negative current corresponds to cooling.)
